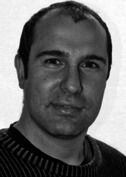# Recent Visible Light and Metal Free Strategies in [2+2] and [4+2] Photocycloadditions

**DOI:** 10.1002/ejoc.202100518

**Published:** 2021-06-10

**Authors:** Marina Sicignano, Ricardo I. Rodríguez, José Alemán

**Affiliations:** ^1^ Organic Chemistry Department Módulo 1 Universidad Autónoma de Madrid 28049 Madrid Spain; ^2^ Institute for Advanced Research in Chemical Sciences (IAdChem) Universidad Autónoma de Madrid 28049 Madrid Spain

**Keywords:** Energy transfer, Metal free, Photocycloaddition, Single electron transfer, Visible light

## Abstract

When aiming to synthesize molecules with elevated molecular complexity starting from relatively simple starting materials, photochemical transformations represent an open avenue to circumvent analogous multistep procedures. Specifically, light‐mediated cycloadditions remain as powerful tools to generate new bonds begotten from non‐very intuitive disconnections, that alternative thermal protocols would not offer. In response to the current trend in both industrial and academic research pointing towards green and sustainable processes, several strategies that meet these requirements are currently available in the literature. This Minireview summarizes [2+2] and [4+2] photocycloadditions that do not require the use of metal photocatalysts by means of alternative strategies. It is segmented according to the cycloaddition type in order to give the reader a friendly approach and we primarily focus on the most recent developments in the field carried out using visible light, a general overview of the mechanism in each case is offered as well.

## Graphical abstract

This Minireview seeks to highlight modern advances in the understanding of novel methodologies that open access to visible‐light‐mediated/metal free [2+2] and [4+2] cycloadditions. A collection of examples that displays reactivity and fundamental contributions in this fast‐evolving field of metal‐based photocatalysis; and could be used as a tool for the readers, to adopt as a kaleidoscopic perspective on cycloadditions.

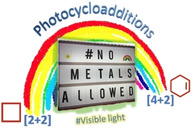

## 1 Introduction

**Scheme 1:**
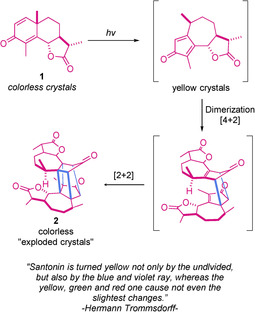
First photochemical observation by Hermann Trommsdorff.

It is well known that photoinduced reactions offer efficient strategies when crafting organic frameworks that might be difficult to access by summoning thermal procedures.^[1]^ This well‐deserved attractiveness of photochemical transformations derives from the opportunity to convert simple feedstocks into highly functionalized molecules in a relatively reduced number of steps. In this sense, cycloaddition reactions shine as a minimalistic tool, by offering the possibility to create multiple bonds and even stereocenters in one single step with predictable stereochemistry.^[2]^ The use of UV light as energy source for promoting these type of transformations lies in the literature since the beginning of the 19^th^ century, when Hermann Trommsdorff discovered in 1834 that natural product santonin **1** reacted upon sunlight exposure through a puzzling series of cycloadditions (Scheme 1).^[3]^ Since then, a great variety of fascinating protocols highlighting the power of UV light for the assembly of polycyclic carbon scaffolds has aroused. However, in response to the current trends for replacing UV irradiation for visible‐light techniques, the design and use of photocatalysts containing visible‐light chromophores has aggressively evolved.^[4]^ Loosely explained, the excited state of a determined photocatalyst might serve as single electron acceptor via reductive quenching, as single electron donor via oxidative quenching or merely as photosensitizer via energy transfer. Unsurprisingly, this versatile behavior of photocatalysts has unlocked diverse approaches to successfully carry out beautiful photocycloadditions. Interestingly, a major part of these examples uses metallic photocatalysts, namely Ir‐ or Ru‐centered, and has been compiled in several delighting reviews.^[5]^ As a complementary work, this review aims to recontextualize the state‐of‐the‐art related to photocycloadditions, by presenting interesting strategies which elude the use of metal‐based photocatalysts under visible‐light conditions.

## 2 [2+2] Cycloaddition. Crafting Four Membered Rings

### 2.1 Fundamental considerations

**Scheme 2:**
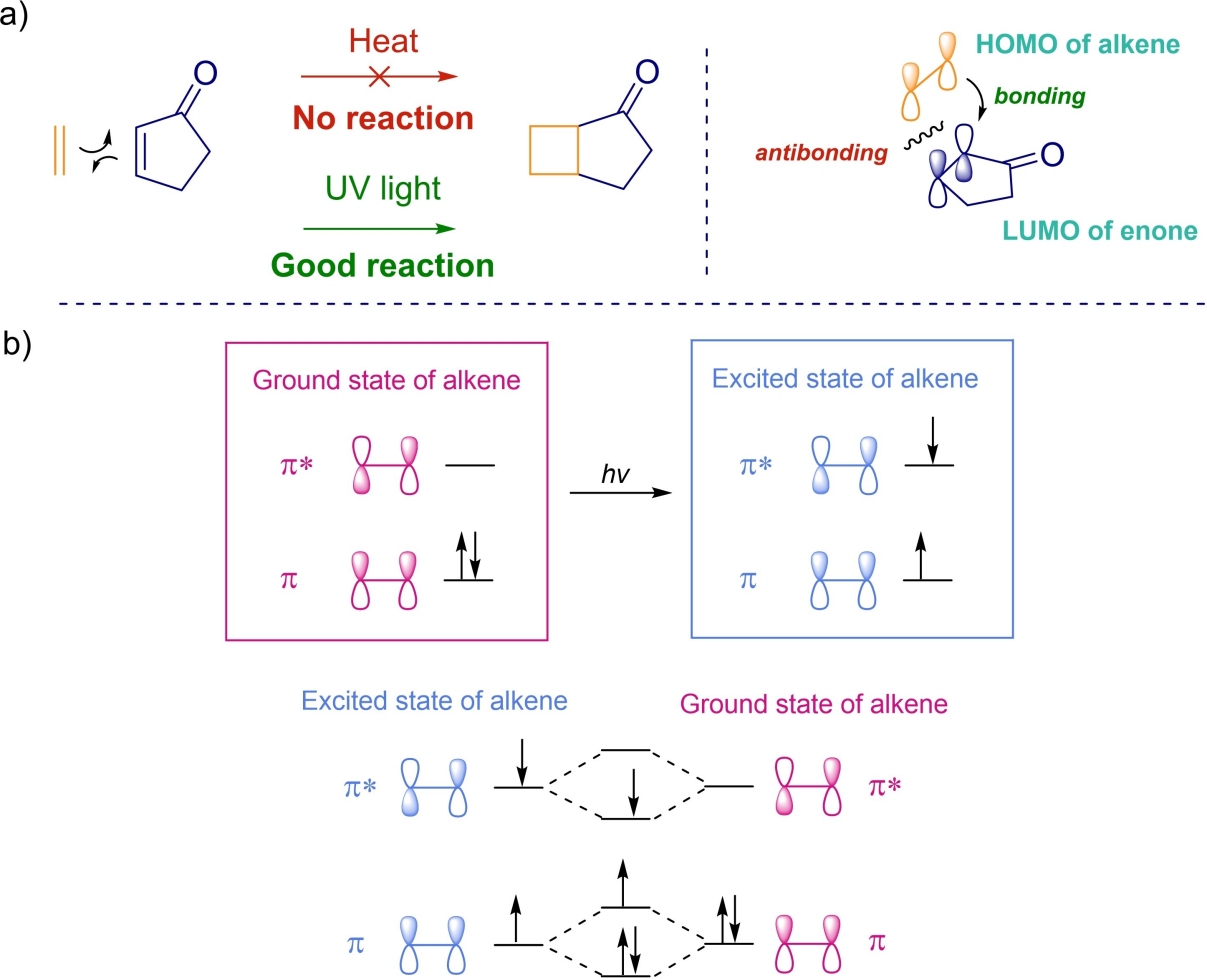
General considerations of [2+2] photocycloadditions.

Among all kinds of organic reactions, pericyclic ones are the most tightly controlled by orbitals, and having a deep understanding of its frontier orbital description is always useful for the development of new ideas.[^6^, ^7^, ^8^] In a common cycloaddition two new bonds are formed at the same time, this requires arranging two filled *p* orbitals and two empty *p* orbitals in order to promote availability in terms of place and right symmetry. As depicted in Scheme 2a, when trying to build the corresponding cyclobutane product from the HOMO (π) of the alkene and the LUMO (π*) of the double bond in the cyclopentenone (under thermal conditions), no cycloaddition reaction occurs because of the antibonding disposition at one end. This symmetry incompatibility problem is avoided by converting one of the double bonds into its excited state photochemically. Upon light irradiation, it is possible to take one electron from the π to the π* orbital (Scheme 2b), and thus by the combination of the excited state of one alkene with the ground state of a second one, the problem is solved: three electrons go down in energy while only one goes up. Following this logic, alkenes may undergo photodimerization, however, reactions between two different scaffolds are obviously more interesting and in order to achieve this in a predictable fashion, it is desirable to reach relatively long‐lived excited states.

**Scheme 3:**
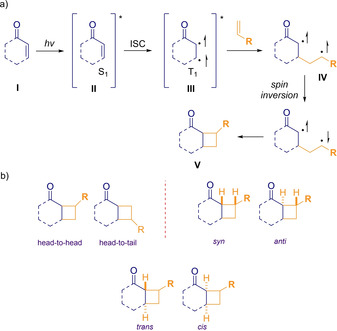
Photocycloaddition via excited triplet state (*T_1_*) **III**.

This latter prerequisite is often fulfilled by using carbonyl‐conjugated olefins. Classic strategies invoke enones as starting materials[^9^, ^10^, ^11^, ^12^, ^13^, ^14^, ^15^] because the relatively easiness to get excited to the singlet state (S_1_), from which evolves to its respective triplet state through intersystem crossing (ISC). At this stage, it is reached the lowest‐lying triplet state (T_1_), which turns to be the starting point of several photochemical transformations (Scheme 3a). In addition to this information, it is important to stay aware of the plausible isomers that one could expect from this type of transformations and to be familiarized with the proper nomenclature when describing the relative disposition of the substituents in the new cyclobutane moiety (Scheme 3b).

**Scheme 4:**
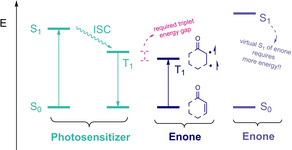
Photocycloaddition by triplet energy transfer.

Moreover, it is worth to note that reaching this triplet state is not limited to direct excitation of the *α*,*β*‐unsaturated carbonyl system, but also by energy transfer from another photoexcited molecule. This process is known as triplet energy transfer or photosensitization,^[16]^ (Scheme 4) and results crucial to keep this in mind for the aims of the present review, as typical excitation wavelength of enones oscillates between 300–380 nm, below the visible light range. In other words, this strategy results as a clear advantage when designing novel visible‐light transformations against direct photoexcitation of unsaturated carbonyl systems,^[17]^ since this latter would require irradiation by a more energetic wavelength.

**Scheme 5:**
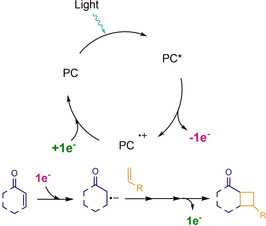
Representative photocycloaddition mediated by SET from a catalyst to an unsaturated system.

Alternatively, it is also possible to start a [2+2] cycloaddition by means of a photoinduced electron transfer (PET).[^18^, ^19^, ^20^, ^21^] This strategy involves the reduction or oxidation of the olefin in matter by the catalyst or an external electron transfer reagent, and not by the excited singlet or triple state of the starting material. In Scheme 5, it is shown a representative case of reductive quenching, where the enone is reduced and the resulting radical anion adds inter‐ or intramolecularly to a second alkene. In order to regenerate the photocatalyst, a back‐electron transfer (BET) process is performed prior or post to the new 4‐membered ring construction. Following an oxidative quenching instead, the olefin would become the respective radical cation, following an analogous sequence of steps prior to BET and delivering the final cyclobutane derivative.

As it will be shown in the following examples, some exceptions are encountered in the literature, which result in enriching the knowledge that currently is handled by chemists.

### 2.2 State‐of‐the art

**Scheme 6:**
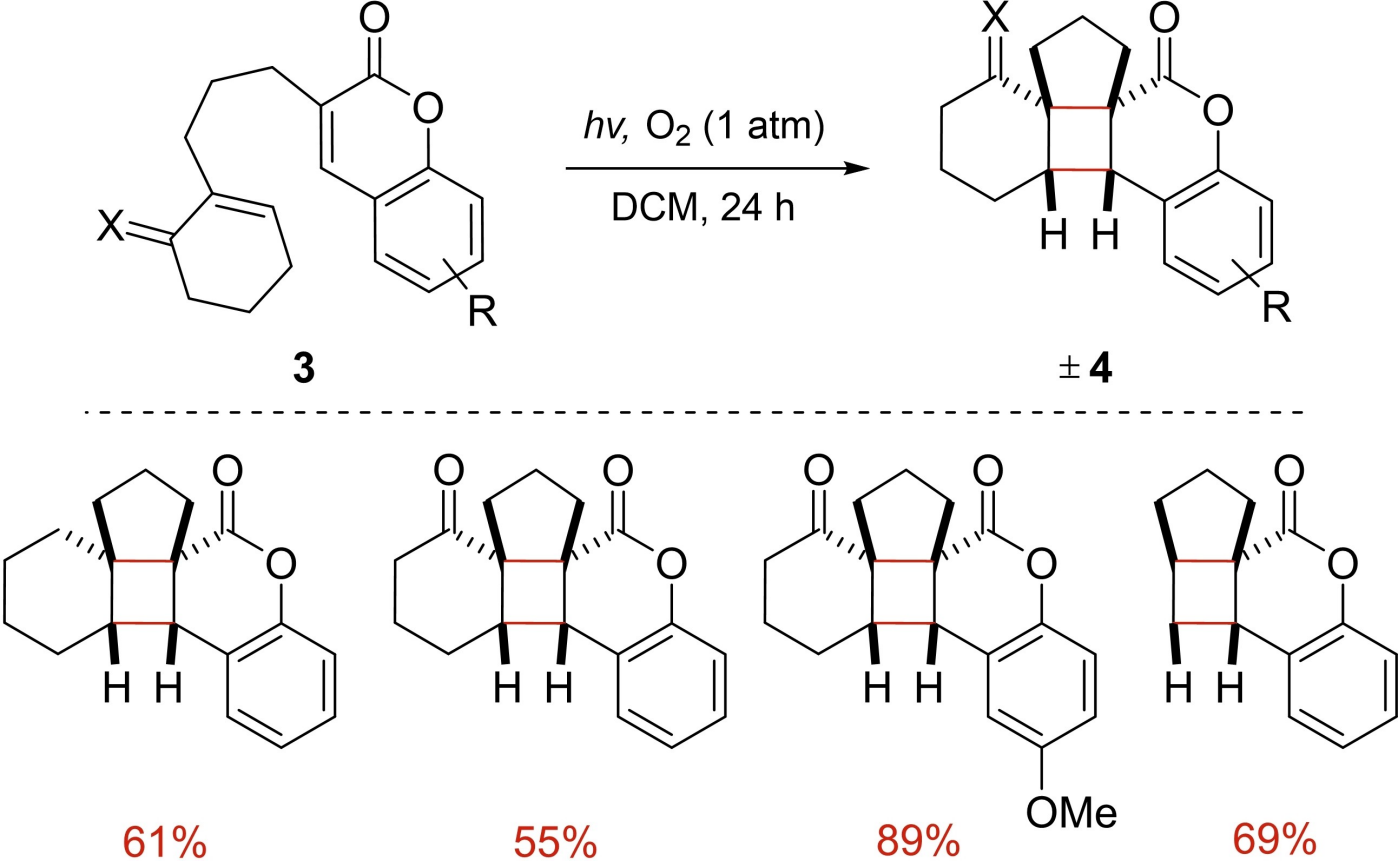
Application of coumarins in intramolecular [2+2] photocycloaddition.

In 2012, Griesbeck and Schmalz introduced the use of *α*,*β*‐unsaturated coumarins as starting materials for intramolecular [2+2] photocycloadditions (Scheme 6).^[22]^ During the investigations, they realized that degassed solvents afforded low yields compared to non‐degassed ones rationalizing that the presence of oxygen might be involved in the reaction mechanism. Indeed, oxygen proved to play a crucial role in the overall transformation, being the principal quencher of the excited triplet‐state coumarin. The strategy does not require external photosensitizers, as the coumarin skeleton is the photoexcited species under white light irradiation.

**Scheme 7:**
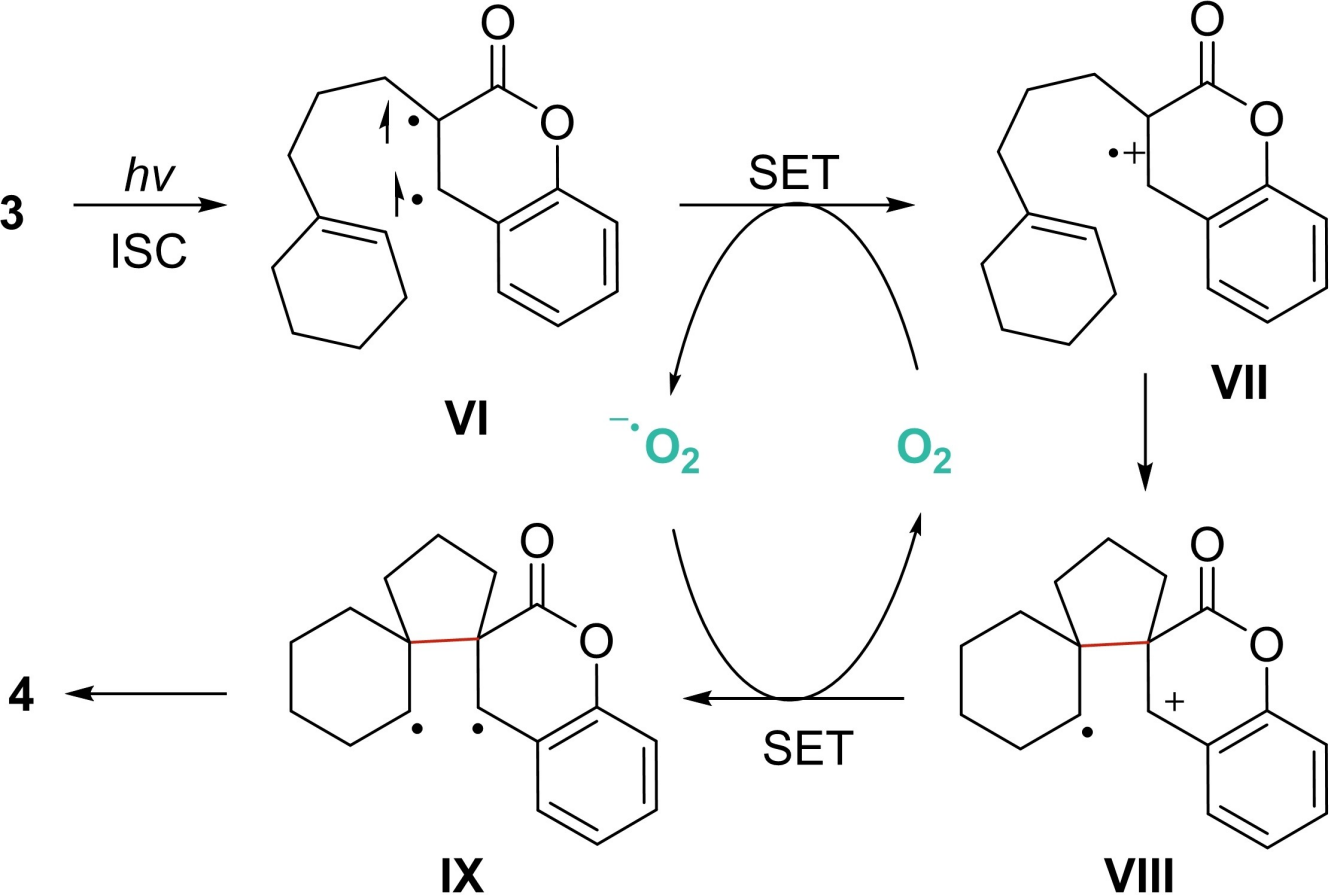
Proposed mechanism for the synthesis of compounds **4**.

After performing several control studies, laser flash photolysis experiments and cyclic voltammetry measurements, a plausible mechanism was suggested. The initial formation of triplet transient **VI** upon light absorption of coumarin **3** and ISC is followed by a single electron transfer (SET) with ^3^O_2_. Then, the resulting radical cation **VII** undergoes a *5‐exo‐trig* radical cyclization to generate distal radical cationic intermediate **VIII**. Afterwards, this latter is reduced by superoxide radical anion leading to biradical IX. Lastly, the desired cyclobutane core is formed by radical coupling delivering photoadduct **4** (Scheme 7).

**Scheme 8:**
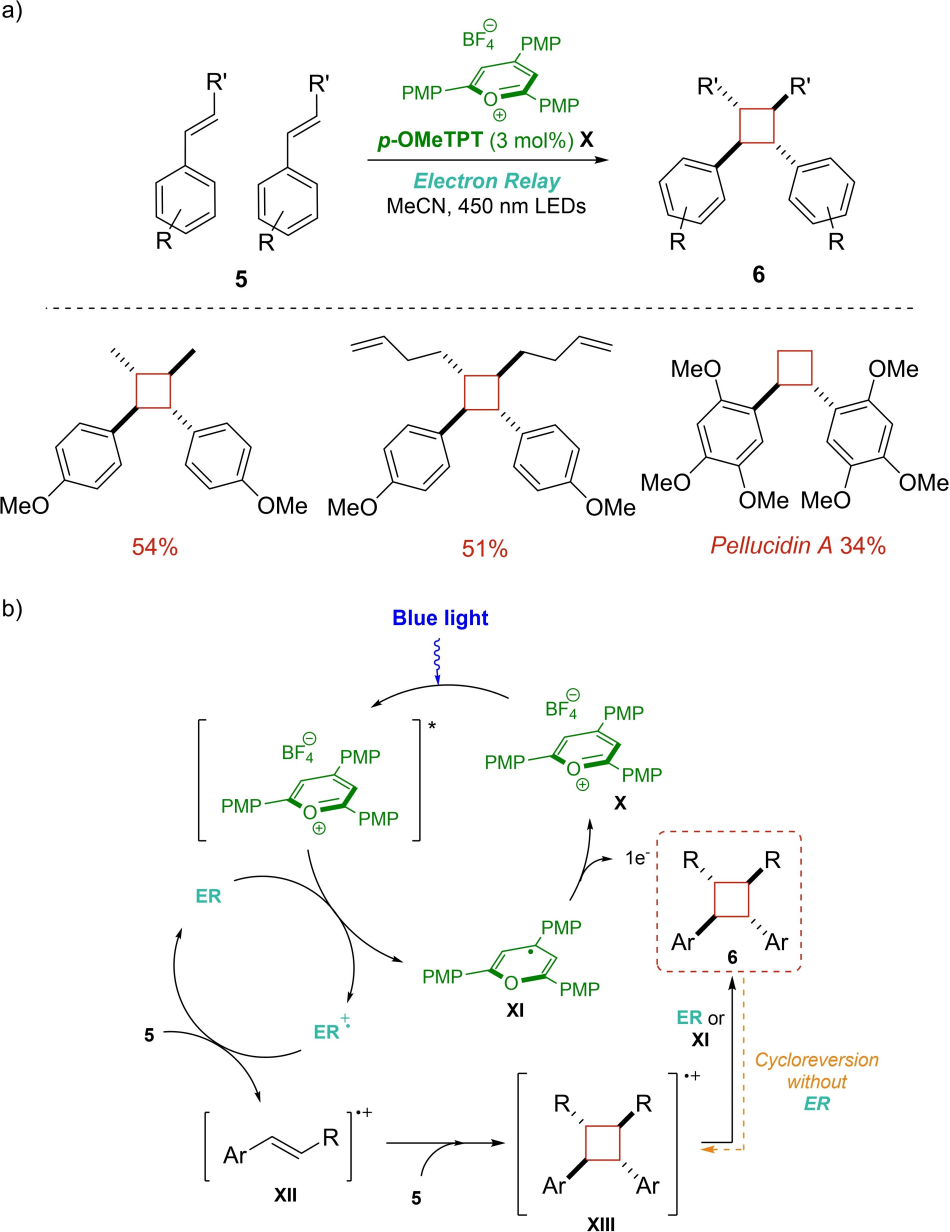
a) [2+2] dimerization of di‐ and monosubstituted alkenes via PET; b) Mechanistic proposal of the electron relay system.

One year later, Nicewicz opened a new synthetic gate to *C_2_*‐symmetric cyclobutane adducts **6** via cyclodimerization of styrene‐derivatives **5**, which allowed them to prepare a family of attractive compounds that are known for their use in antifungal‐ and anticancer drug development (Scheme 8a).^[23]^ A key for the success of the procedure relies in the use of an “electron relay” (ER) (naphthalene, anthracene, or propylene oxide) as quencher of the organic photooxidant (*p*‐MeOTPT)*, as it enables the execution of the reaction under 450 nm LEDs and avoids the use of ultraviolet light. This latter is worth to note considering the fact that, until that moment, classic methodologies were strongly hampered by alkene isomerization, exclusive isolation of *meso* isomers and cycloreversion of the final products. On the basis of the obtained results, the authors suggest that after initial excitation of *p‐*MeOTPT, oxidation of the electron relay (which was carefully selected in order to have a considerably higher oxidation potential than **5**) would conduct to intermediate **XI** (Scheme 8b). After cycloaddition with another molecule of 5, radical cation **XIII** is reduced either by **XI** or the electron relay. Additionally, it was demonstrated that in the absence of the ER, cyclobutanes are prompt to get oxidized, leading to an undesirable cycloreversion.

**Scheme 9:**
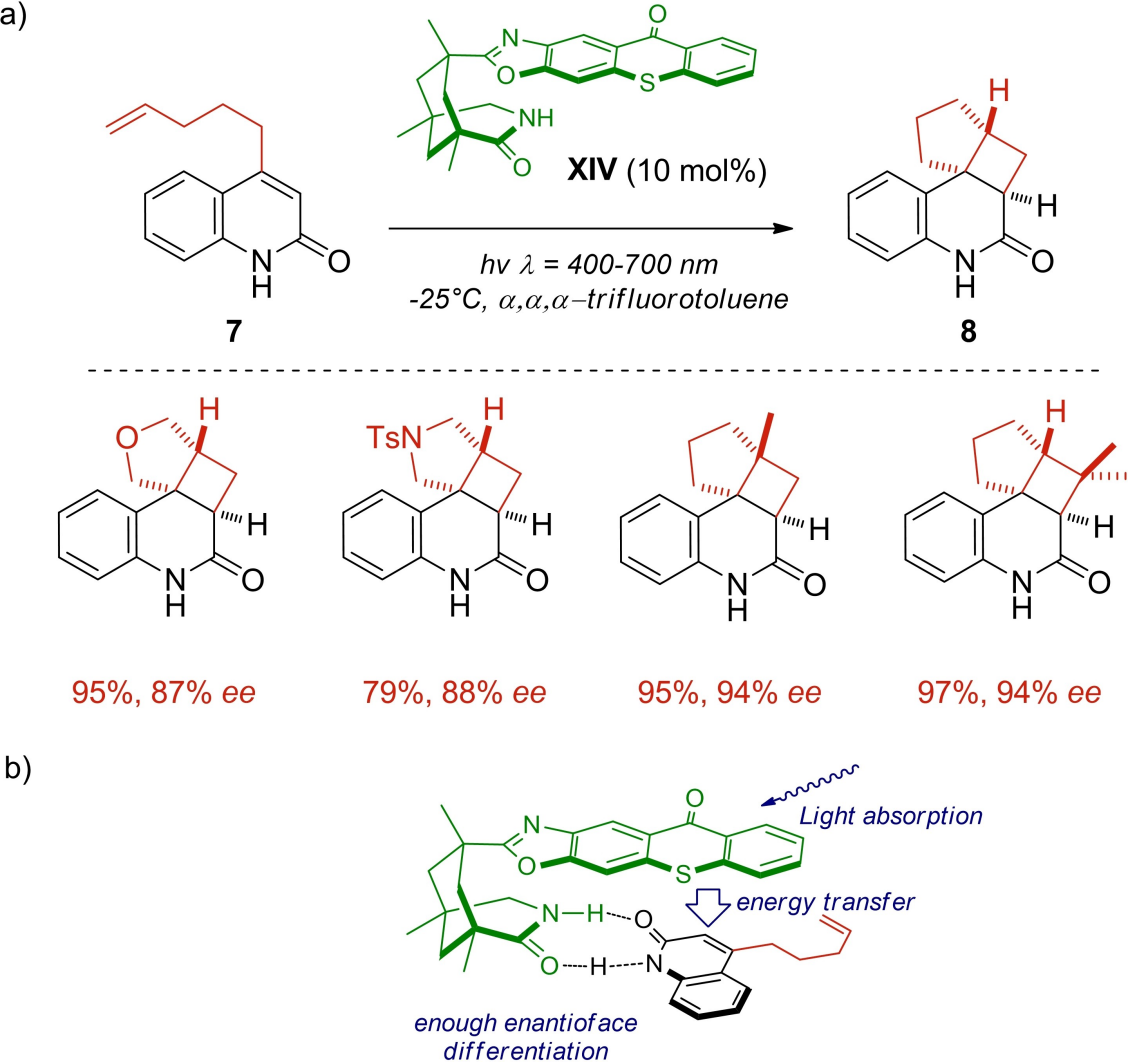
a) Introduction of thioxanthone **XIV** as chiral photocatalyst, b) mechanistic model for sensitization and chiral control.

Later on, the group of Bach described what would become a pivotal example in metal‐free photocatalysis: the chiral thioxanthone motif **XIV** which is capable to absorb visible light exhibiting a classic photocatalyst performance through triplet‐energy sensitization.^[24]^ After studying the spectral properties of the new template and the starting quinolones **7**, the authors were able to prepare the cyclobutane derivatives **8** under 420 nm wavelength irradiation (Scheme 9a). The fact that only unsuccessful results were obtained when suppressing **XIV**, was only delightful for the authors, confirming a “light‐harvesting antenna” behavior which transfers the absorbed energy to the quinolone, presumably by triplet‐energy transfer. Lastly, the recorded high levels of enantioselectivity were addressed to the hydrogen‐bonding properties of the architecture of **XIV**, to which the quinolone substrate is fixed enough to achieve a good enantioface differentiation (Scheme 9b).

**Scheme 10:**
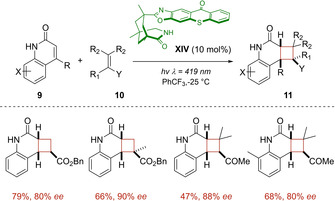
a) Thioxanthone **XIV** as chiral photocatalyst for the [2+2] cycloaddition of quinolones **9**.

The strong ability of photosensitizer **XIV** to accept and donate hydrogen‐bonding simultaneously, was subsequently harnessed by the same group to mediate the enantioselective intermolecular [2+2] cycloaddition of several quinolones **9** and alkenes **10**.^[25]^ The binding between the catalyst and the substrate resulted again to be crucial for the success of the transformation. Indeed, the dissociation of the triplet excited heterocycle 9* from the excited complex, was the responsible of the loss of enantioselectivity, as encountered for the electron‐rich vinyl acetate. In presence of electron‐deficient alkenes 10, good to high regio‐, diastereo‐ and enantioselectivity were smoothly obtained (Scheme 10).

**Scheme 11:**
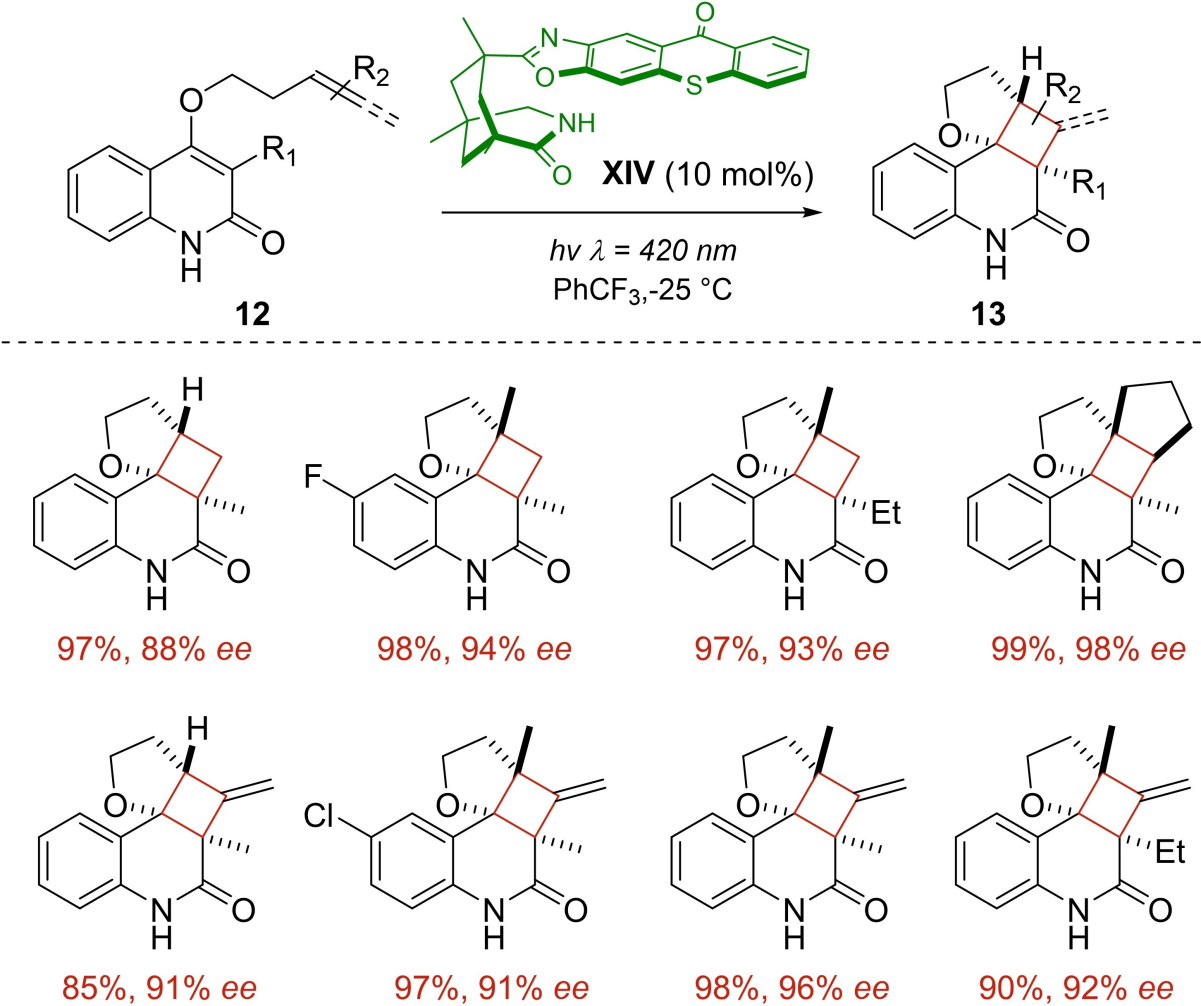
a) Thioxanthone **XIV** as chiral photocatalyst for the intramolecular [2+2] cycloaddition of 3‐alkylsubstituted quinolones **12**.

Due to the increasing interest in the formation of highly substituted chiral cyclobutanes in a relatively simple manner, the group of Bach investigated the reaction of several 3‐alkylsubstituted quinolones **12** including an alkene or allene moiety tethered to the C4 carbon atom via an alkoxy linker. Under visible light irradiation, the organocatalyst **XIV** efficiently photosensitized the intramolecular [2+2] cycloaddition enabling the efficient synthesis of the intriguing products **13** with high enantioselectivity (Scheme 11).^[26]^

**Scheme 12:**
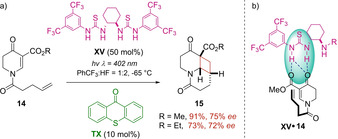
a) Bisthiourea **XIV** as chiral organocatalyst b) Mechanistic model of the complex **XV** **14**.

Another activation mode for the construction of enantioenriched cyclobutane products was explored by the group of Bach as well. In presence of thioxanthone (TX) as triplet sensitizer, chiral bisthiourea **XV**, derived from the commercially available (1*R*,2*R*)‐diaminocyclohexane, was able to induce enantioface differentiation through the establishment of multiple hydrogen‐bonding associations with the two tested substrates (Scheme 12b). The corresponding products **15** were obtained with good yields and enantioselectivity (Scheme 12a). Despite the limited scope that was executed, this investigation works as a proof of concept related to the plausibility to use non‐covalent interactions as tool for stereocontrolled photocycloadditions.^[27]^

**Scheme 13:**
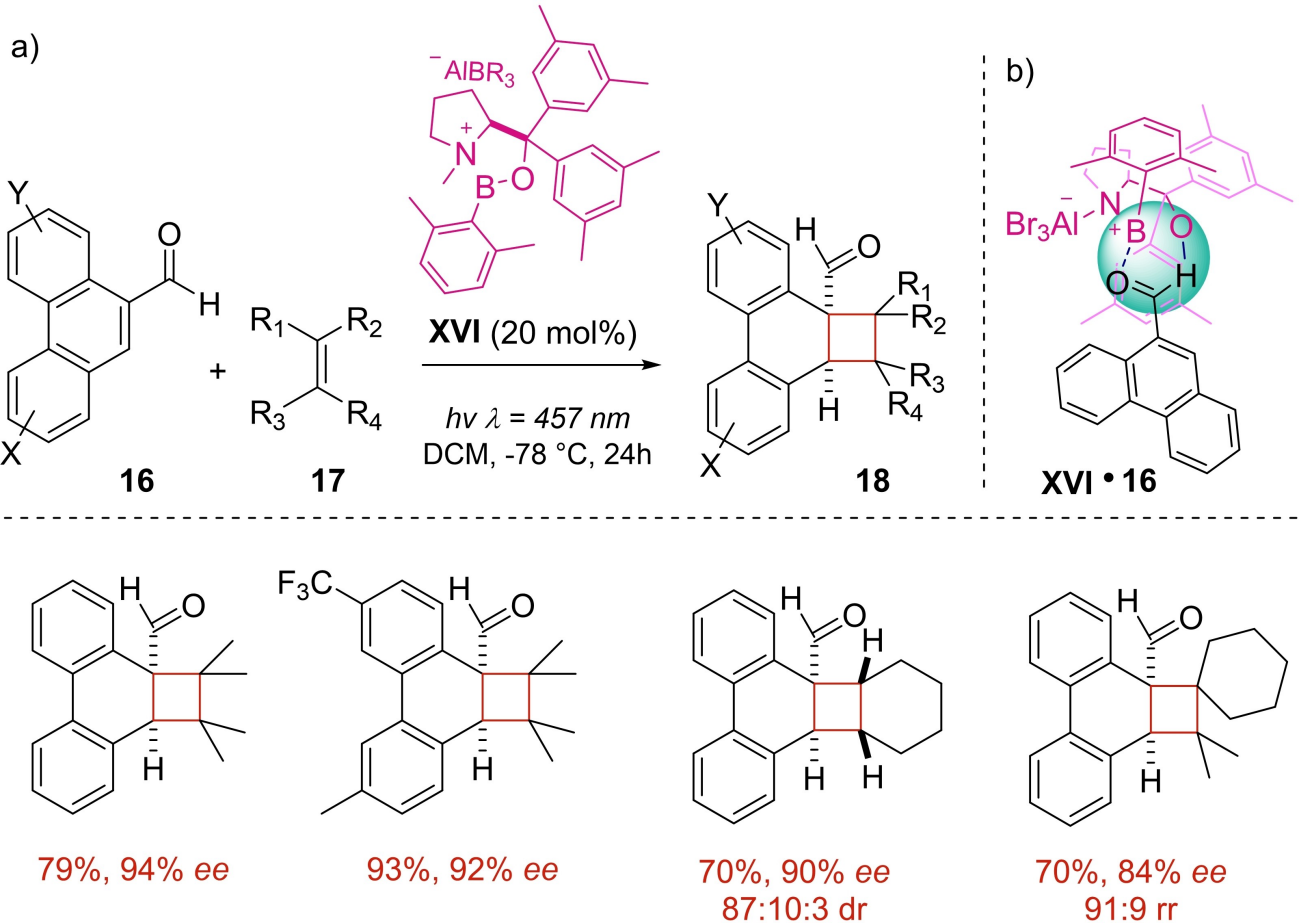
a) Oxazaborolidine Lewis acid **XVII** as chiral organocatalyst b) Mechanistic model of the complex **XVI** **16**.

Two years later, the same research group demonstrated that chiral oxazaborolidine Lewis acid **XVI** flawlessly worked as good organocatalyst for the enantioselective [2+2] photocycloaddition between phenanthrene‐9‐carboxaldehydes **16** and olefins **17**.^[28]^ Thanks to the coordination of the carbonyl group to the boron atom and by a non‐classical hydrogen bonding (Scheme 13), a high enantioface differentiation was achieved, favoring the preferential attack of the olefin from the less shielded *Si* face of the complex catalyst‐aldehyde **XVI** **16**. This coordination model was also responsible of a bathochromic absorption shift, which induces a selective excitation of the complex **XVII** **16** at long wavelengths (>420 nm) and the generation of cyclobutanes **18** without the assistance of an external photocatalyst. In detail, the authors claim that the most plausible mechanism occurs via an excited singlet state, affecting the selectivity of the reaction.

**Scheme 14:**
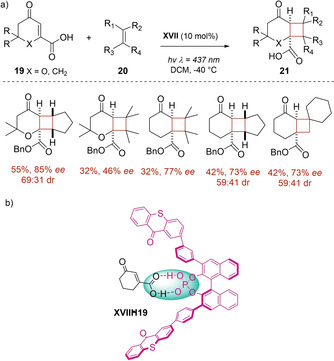
a) Phosphoric acid **XVII** as chiral organophotocatalyst b) Mechanistic model of the complex **XVII** **19**.

Recently, Bach and co‐workers introduced an unprecedented chiral phosphoric acid **XVII** including two thioxantone moieties at the 3,3’ position of the 2,2’‐binaphthol core as new photosensitizer for cycloaddition reactions.^[29]^ Actually, this new phosphoric acid **XVII** showed to efficiently catalyze the asymmetric intermolecular [2+2] photocycloaddition of β‐carboxyl‐substituted cyclic enones **19** leading to the corresponding products **21** with good enantioselectivity (Scheme 14a). As suggested by DFT calculations and NMR studies, the binding between the carboxylic acid and the photocatalyst (Scheme 14b), which occur through the establishment of two hydrogen‐bonding points, should induce a proper enantioface differentiation and, at the same time, lower the triplet energy of the substrate in order to enable the energy transfer mechanism.

**Scheme 15:**
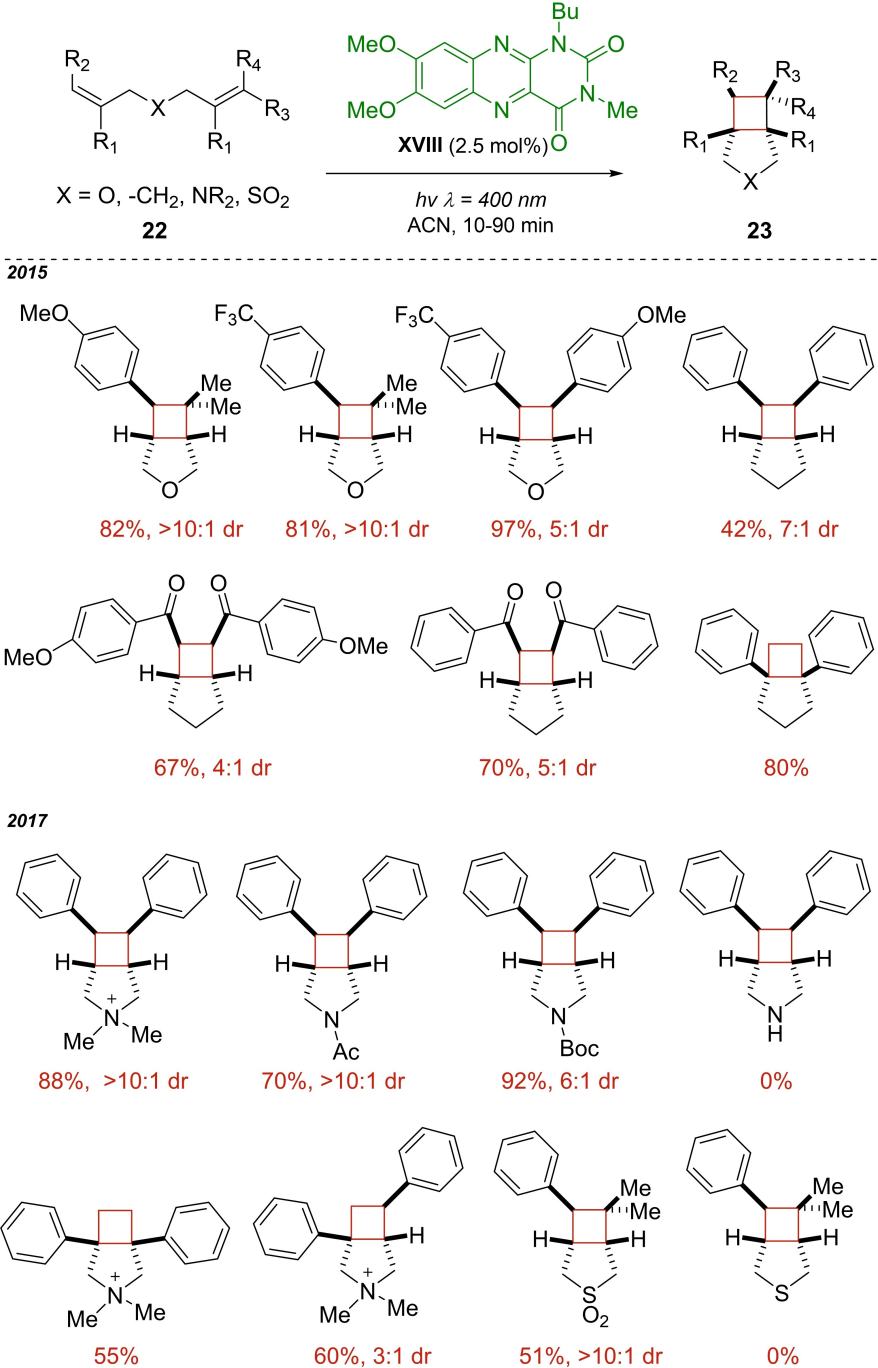
Flavin **XVIII** as photosensitizer for intramolecular [2+2] cycloaddition.

In 2015, Cibulka and co‐workers pioneered the use of flavin derivatives as photocatalysts for the formation of C−C bonds, developing an efficient intramolecular [2+2] photocycloaddition of dienes **22**; until that time, flavins had been exploited only in photooxidation reactions. In this work,^[30]^ the key was the design of tailoring alloxazine **XVIII** able to absorb the light on the border of visible‐light (absorption maximum to approximately 400 nm) and with a high triplet energy (E_T_ ≈245 KJ mol^−1^) that gave it the ability to work as triplet energy sensitizer of the substrate **22** (*E*_T_ ≈249 KJ mol^−1^ for the β‐methylstyrene). Indirect evidence of the proposed mechanism was the detection of triplet state *E*/*Z* photoisomerization of the styrene **22** as well as the generation of cycloadduct products **23** with the same relative configuration regardless of the configuration of the starting materials. Overall, this protocol worked well with a broad range of dienes **22** including both electron‐rich styrene dienes and electron‐poor bis(arylenones), producing cyclobutanes **23** quantitatively and with high *exo*‐*cis*‐diastereoselectivity (Scheme 15). Two years later, this protocol was successfully extended to several aza‐1,6‐dienes **22** protected by acylation or quaternization for the synthesis of phenyl‐ and diphenyl‐3‐azabicyclo[3.2.0]heptanes **23**.^[31]^ Also a sulfone derivative underwent an analogous cyclisation as reported in Scheme 15.

**Scheme 16:**
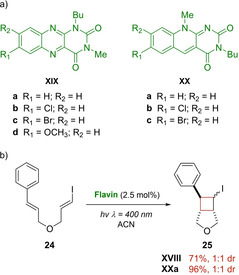
a) Flavins **XVI** and **XVII** as new photocatalysts; b) [2+2] photocycloaddition of iodo‐diene **11**.

Moreover, the same research group, in the 2017, introduced new alloxazine **XIX** and deazaflavin **XX** derivatives (Scheme 16a) to mediate [2+2] photocycloaddition reactions of dienes **22**.^[32]^ Comparing the results already achieved with the catalyst **XVIII**, the new deazaflavins **XX** provide similar or slightly better results with styrene compounds **22**, although in longer reaction times, whereas the novel alloxazines showed to be completely inefficient. When the electron‐poor bis(arylenones) substrates were, instead, investigated, quantitative conversions were achieved in almost all cases. In that article, note of worthy was the [2+2] photocycloaddition of the iodo‐diene **24** promoted by the catalyst **XVIII** and **XXa** as represented in the Scheme 16b.

**Scheme 17:**
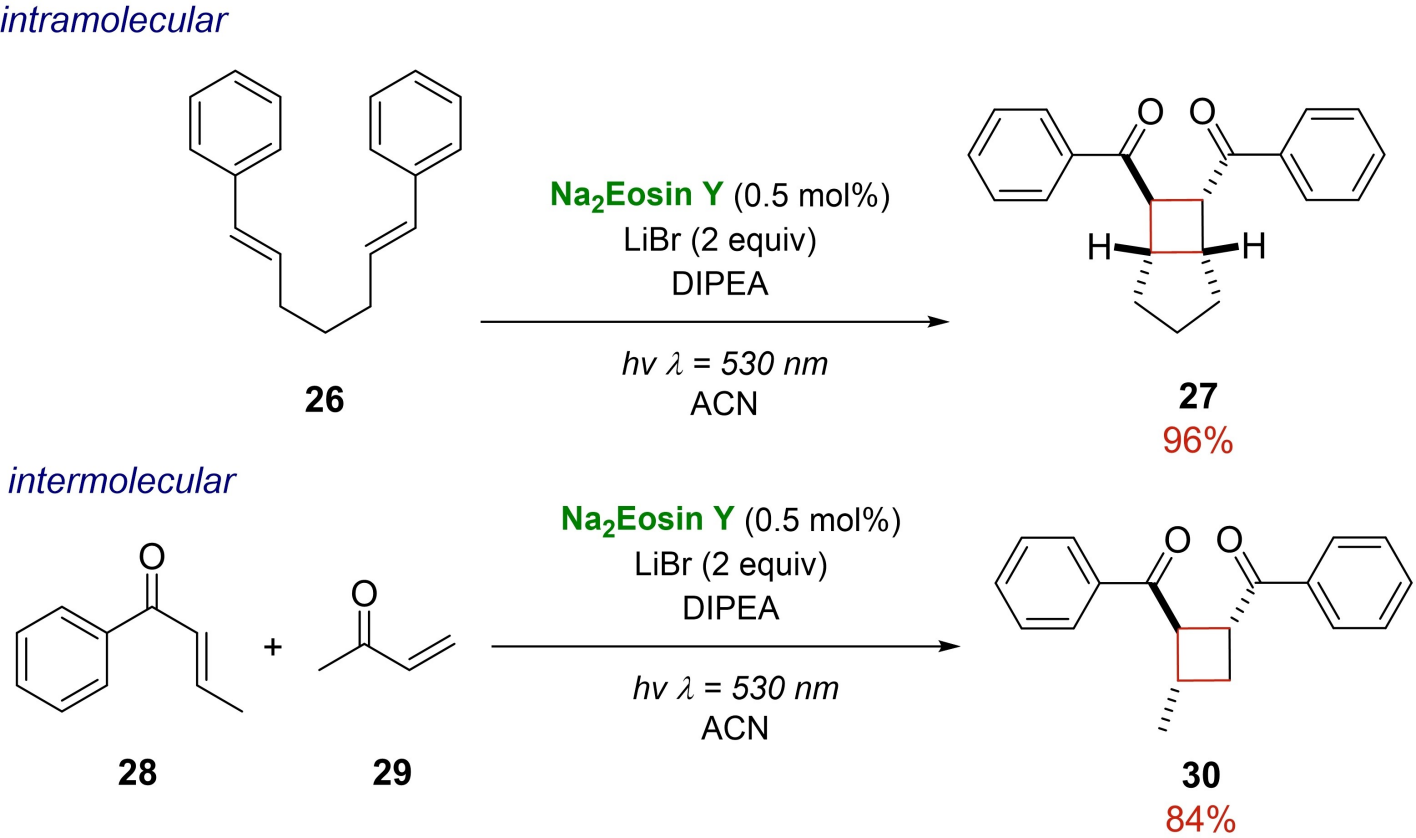
a) Intra‐ and intermolecular [2+2] photocycloaddition promoted by Na_2_Eosin Y.

An example of *trans*‐cyclobutane **27** was reported by Zeitler group, in 2013, using Na_2_Eosin Y as photocatalyst, LiBr as Lewis acid and irradiating with green LED (Scheme 17a).^[33]^ The same conditions were successful applied for the intermolecular [2+2] cycloaddition of (*E*)‐1‐phenylbut‐2‐en‐1‐one **28** with but‐3‐en‐2‐one **29** as reported in Scheme 17b.

**Scheme 18:**
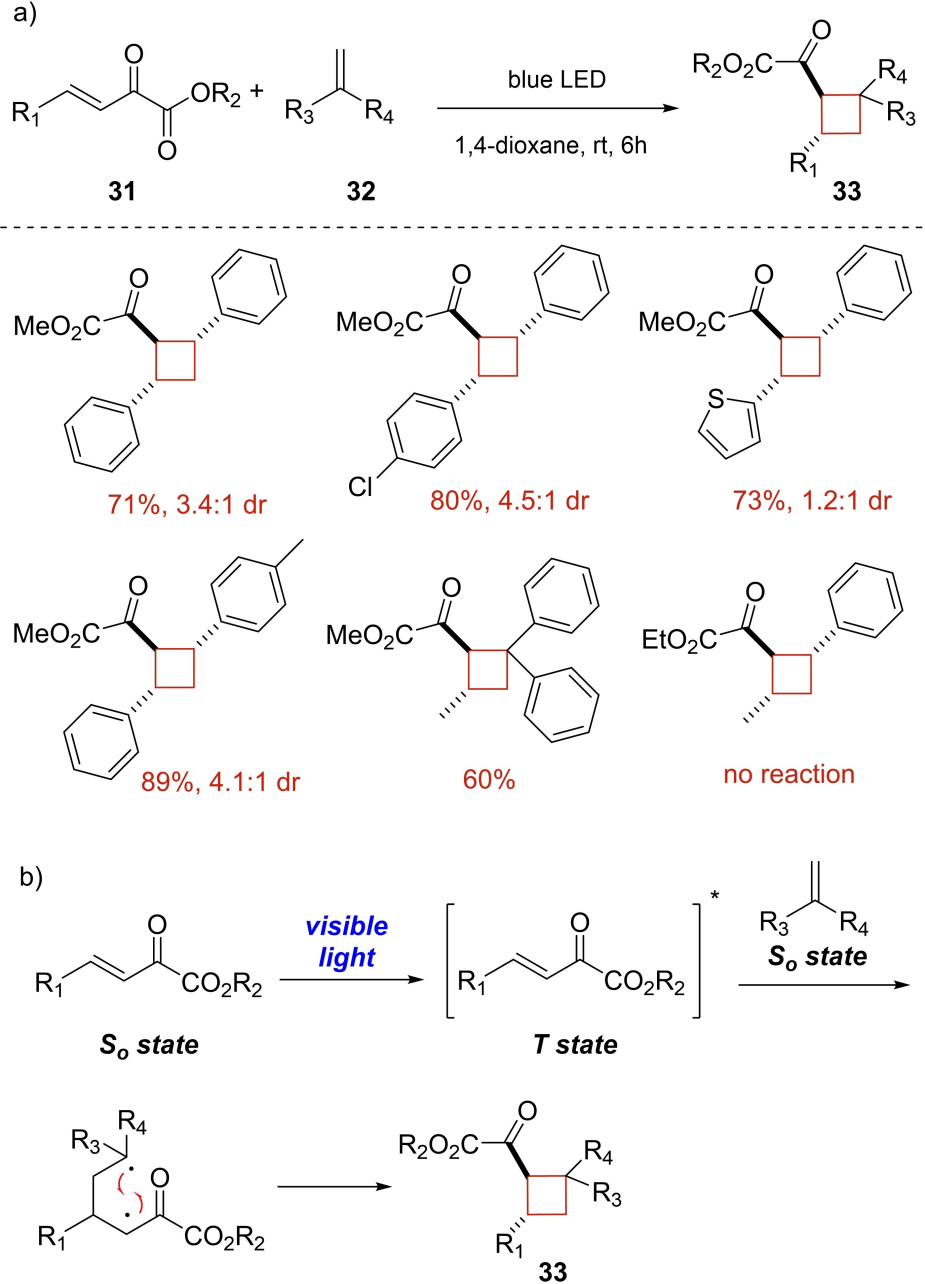
a) Scope of the [2+2] photocycloaddition of **31** with simple olefins **32**; b) Proposed mechanism of the reaction.

In 2017, Luo and coworker encountered that β,γ‐unsaturated α‐ketoesters **31** can adsorb visible light directly achieving their triplet state from singlet state without assistance of any photosensitizer.^[34]^ This observation induce the authors to investigate an interesting catalyst free [2+2] photocycloaddition reaction with simple olefins **32**. As resumed in Scheme 18a, several substituted cyclobutanes **33** with good yields and moderate diastereoselectivity were isolated. Unfortunately, this methodology was not working with aliphatic α‐ketoesters. In the Scheme 18b, the proposed mechanism of the photoreaction is presented.

**Scheme 19:**
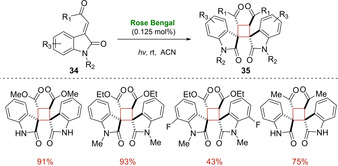
Rose bengal as photocatalyst for intermolecular [2+2] cycloaddition of 3‐ylideneoxindoles **18**.

In the same year, Guan and He introduced 3‐ylideneoxindoles **34** as ideal substrates for the synthesis of diverse spirocyclic compounds **35** via [2+2] photocycloaddition using Rose Bengal as triplet sensitizer.^[35]^ As depicted in Scheme 19, all products were obtained in good yields and with excellent diastereo‐ and regioselectivity (only one diastereomer) by employing a very low catalyst loading. Interestingly, both esters and ketones of the 3‐ylideneoxindoles **34**, as well as *N*‐protected and unprotected heterocycles worked well under the optimized reaction conditions.

**Scheme 20:**
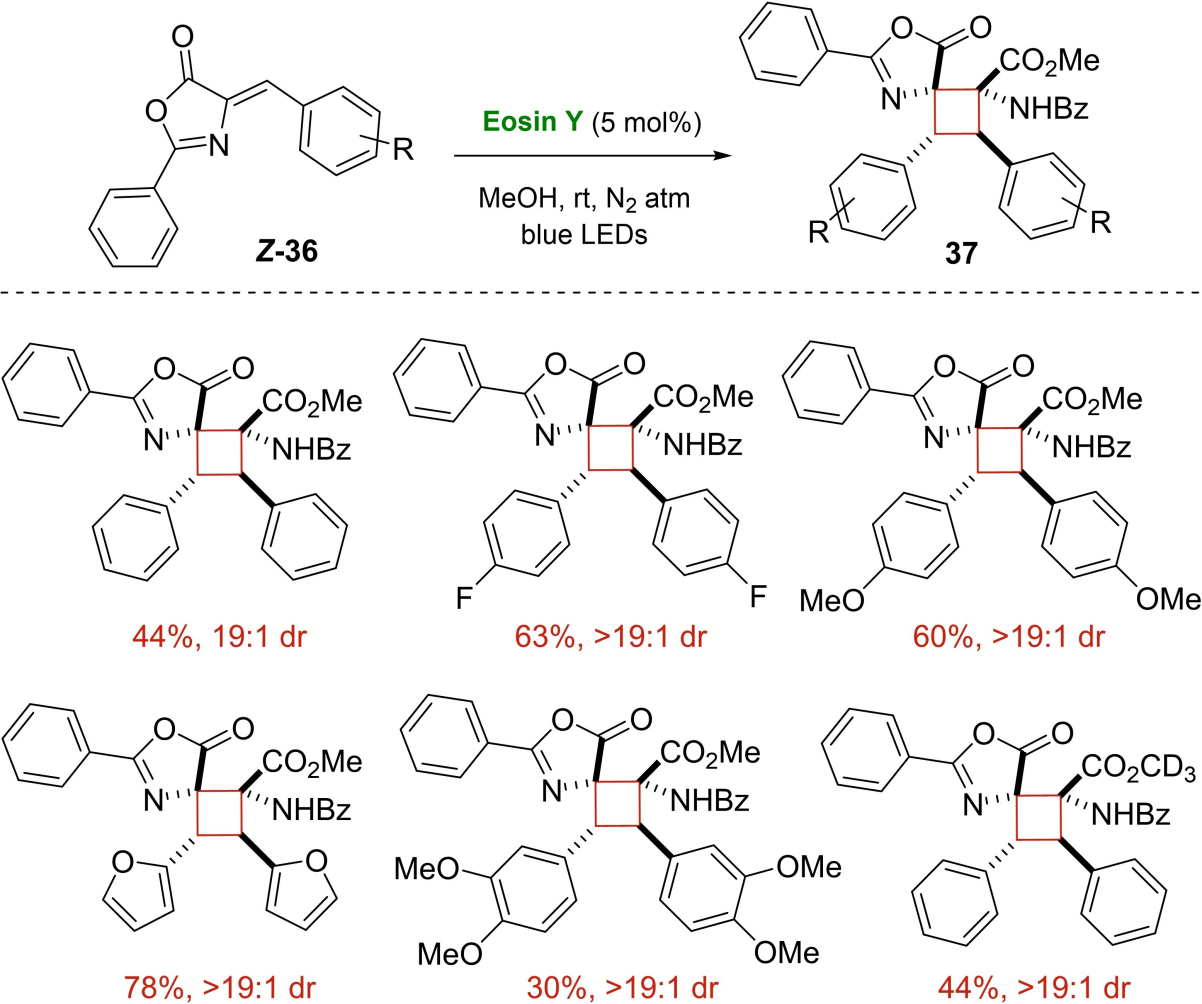
Intermolecular [2+2] photocycloaddition of Erlenmeyer–Plöchl Azlactones

In 2018, the first intermolecular [2+2] photocyclization of Erlenmeyer–Plöchl Azlactones **36** for the synthesis of 1,2‐diaminotruxinic acid derivatives **37** was studied by Amarante group.^[36]^ In an optimized protocol including Eosin Y and blue LED irradiation, various bicyclic spiro‐type cyclobutanes **37** were produced with moderate to good yields and perfect diastereocontrol, achieving just one diastereomer (1,2‐*Z*,*E*‐*anti*) in all cases (Scheme 20).

**Scheme 21:**
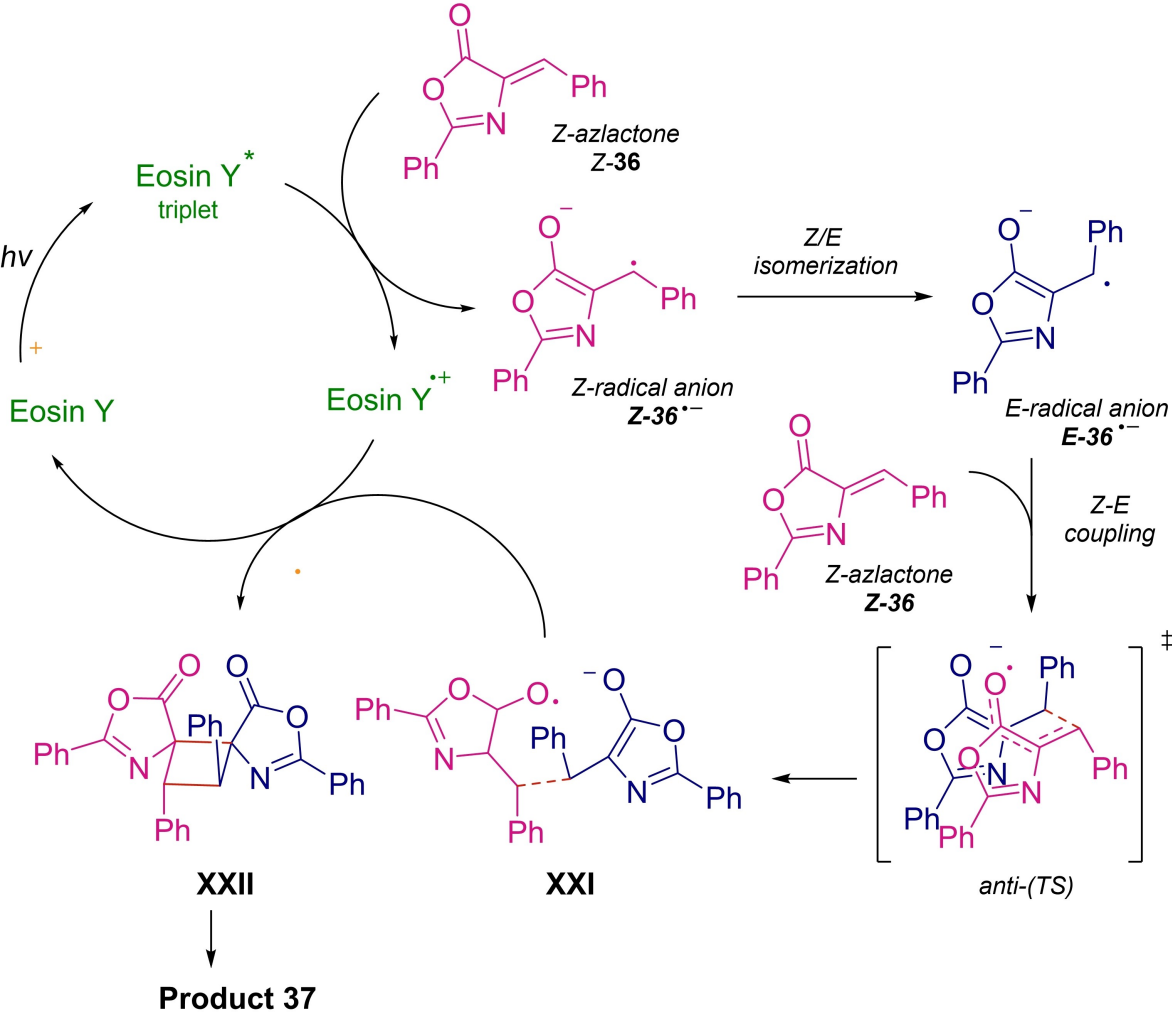
Proposed mechanism of Intermolecular [2+2] photocycloaddition of Erlenmeyer–Plöchl Azlactones.

In order to elucidate the reaction mechanism, the authors conducted accurate DFT analysis. As depicted in Scheme 21, the formation of *Z*‐azlactone radical cation (***Z*** **‐36^+^**), generated after electron transfer with the triplet state of the photocatalyst, occurs. Then, this radical intermediate ***Z*** **‐36^+^** should be inclined to undergo fast *Z*/*E* isomerization, which is inevitable to obtain the *E*‐azlactone radical anion (***E*** **‐36** ^−^) and the subsequent addition to another neutral *Z*‐azlactone molecule. As well argued, this latter step should be the step in which the configuration of the product is determined. In fact, only the *anti*‐coupling between the *E*‐*Z* azlactones seems to be favored, and, thus, justifying the excellent diastereoselectivity reported. Finally, an immediate ring closure of the cyclobutane moiety with formation of the product **37** should take place, regenerating the catalyst (Scheme 21).

**Scheme 22:**
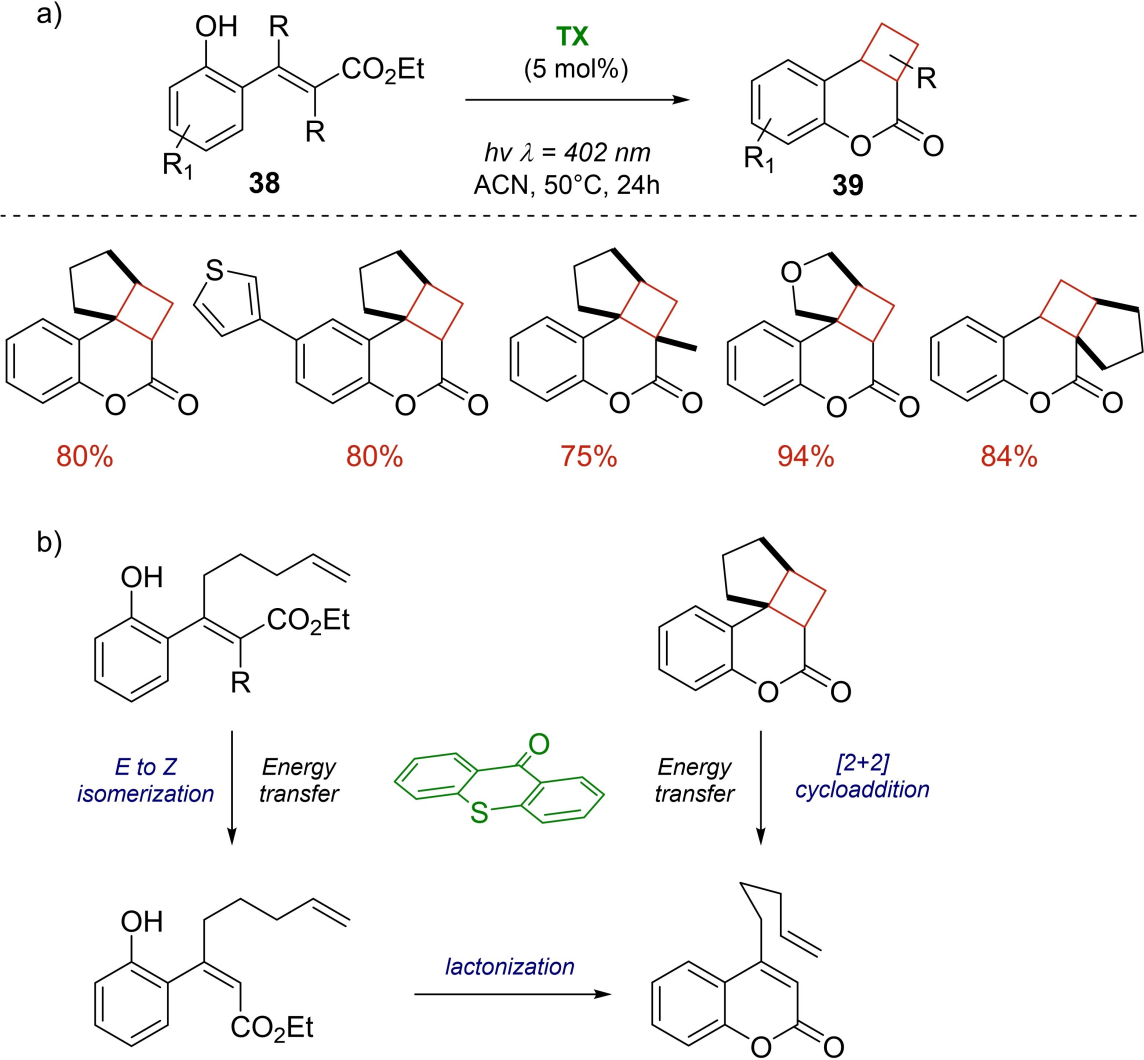
a) TX‐promoted one pot synthesis of angularly‐fused dihydrocoumarins **38**; b) Proposed mechanism of the reaction.

In 2019, Gilmour demonstrated that simple organic photocatalyst as thioxanthen‐9‐one (TX) can enable the generation of complex molecules via sequential π‐bond activation under visible‐light irradiation.^[37]^ Indeed, he developed an interesting one pot cascade reaction for the synthesis of diverse angularly‐fused dihydrocoumarins **23** (Scheme 22) starting from substituted cinnamic acids **22**. As represented in Scheme 22b, this protocol consisted in an *E*‐*Z* alkene isomerization via energy transfer catalysis followed by lactonization and [2+2] cycloaddition. This latter step was again mediated by TX via a second energy transfer event.

**Scheme 23:**
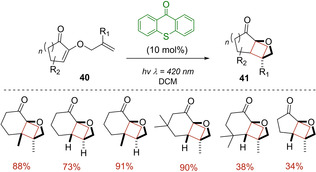
TX‐promoted [2+2] photocycloaddition of 2‐substituted cyclohexenones **40**.

A year later, thioxanthen‐9‐one (TX) was also investigated by Bach as triplet photosensitizer for the [2+2] photocycloaddition of a family of cyclohexenones **40** bearing a 2’‐propenyloxy, 2’‐butenyloxy, 2’‐pentenyloxy or 2’‐methyl‐2’‐propenyloxy group in the 2‐position.^[38]^ Being these shorter tethers, crossed products **41**, rather than straight ones, were achieved in all cases with good yields and high regio‐ and diastereoselectivity. Conversely, the analogous cyclopentenones showed to be less prone to undergo the intramolecular cycloaddition, obtaining the corresponding cyclobutane product just in one case, as represented in Scheme 23.

**Scheme 24:**
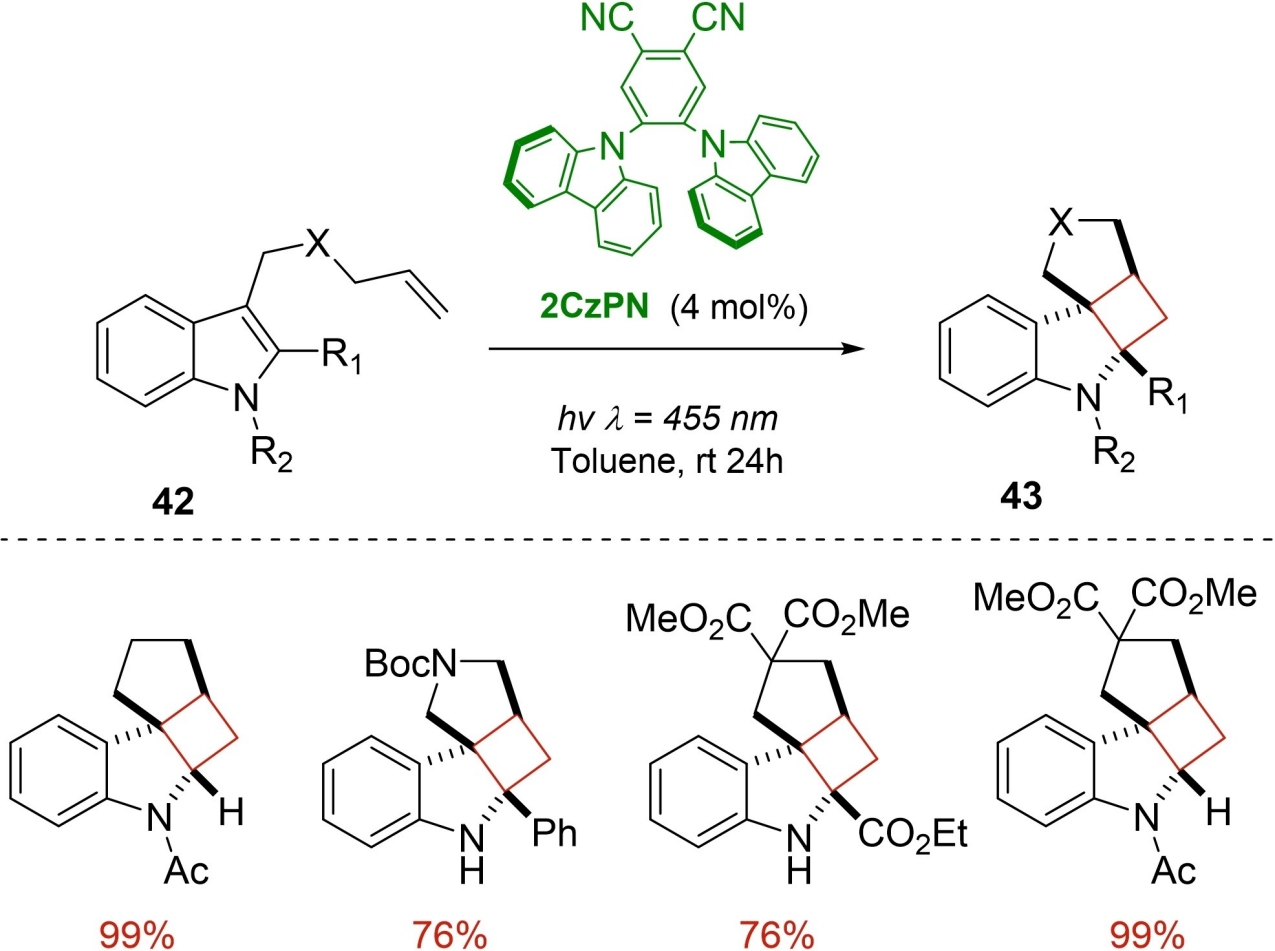
2CzPN‐catalyzed photocycloaddition of indoles **42**.

**Scheme 25:**
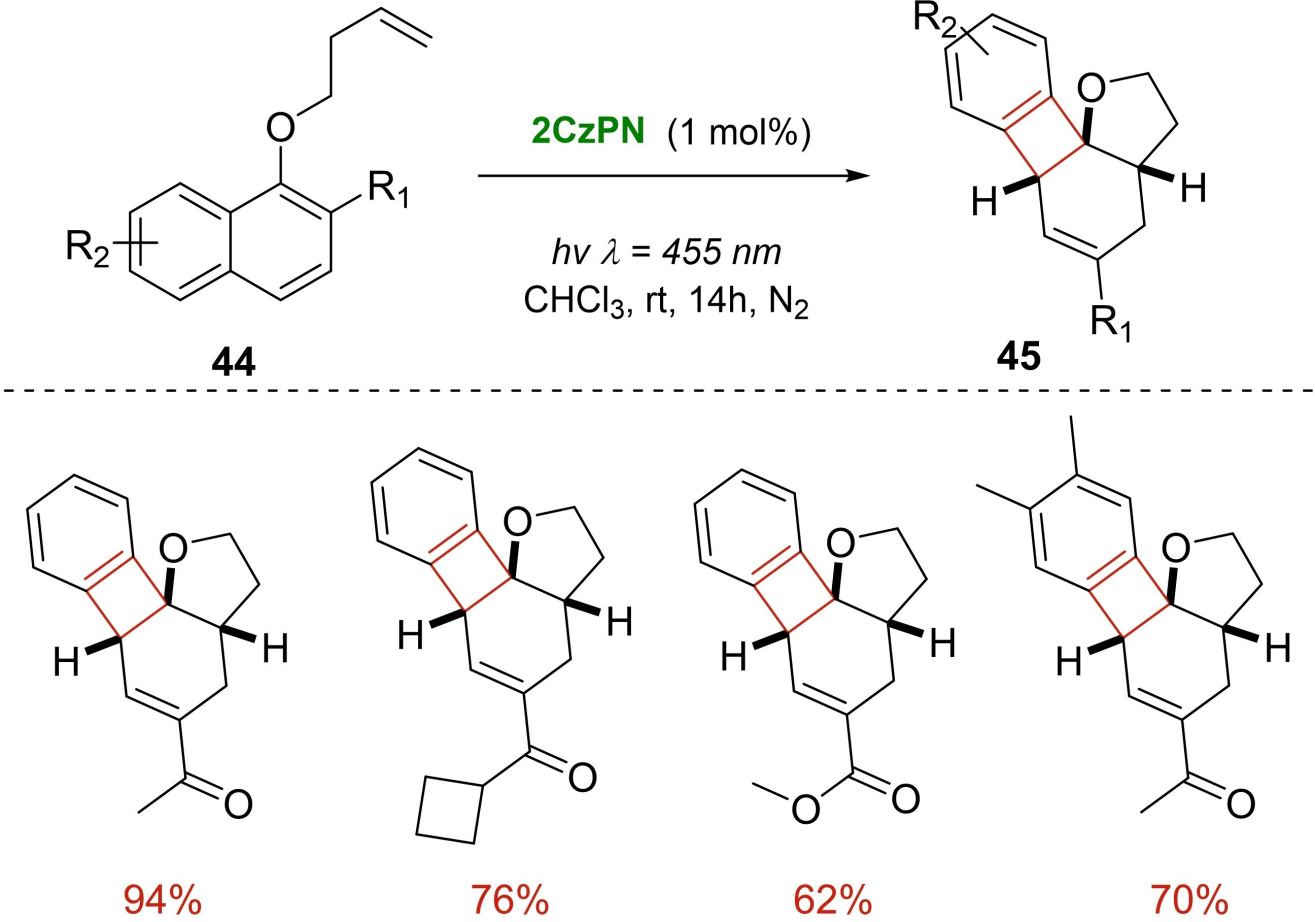
2CzPN‐catalyzed photocycloaddition of naphthols **44**.

Later on, the cheap and organic 1,2‐bis (carbazol‐9‐yl)‐4,5‐dicyanobenzene (2CzPN) was introduced by Koenig with the aim of further extend the scope of visible light dearomative cycloaddition reactions.^[39]^ This triplet‐energy photosensitizer showed to smoothly catalyzed, under blue LED irradiation, [2+2] cycloadditions of both indoles **42** (Scheme 24) and naphthols **44**, giving access to diverse polycyclic compounds with good yields and selectivity (Scheme 25). In particular, in the case of naphthols, the reaction proceeded via two sequential triplet energy excitation pathways promoted by this single organocatalyst. Furthermore, thanks its uncharged nature, 2CzPN displayed a good solubility both in polar and non‐polar solvents also enabling the use of toluene as reaction media. An easy recovery and reuse of the organocatalyst via column chromatography was also claimed by the authors.

**Scheme 26:**
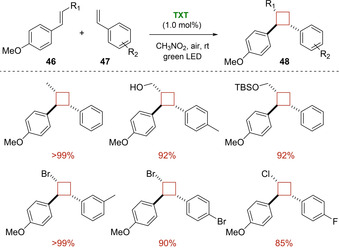
TXT‐catalyzed intermolecular photocycloaddition of styrenes.

An example of single electron transfer intermolecular [2+2] cycloaddition was reported by Honda and co‐workers in 2020.^[40]^ In this work, a selective oxidation of electron‐rich β‐substituted styrenes **46** such as *trans*‐anethole and β‐bromostyrene over simple styrenes **47** was successfully achieved thanks to the unique reduction potential (*E*_0_’(C^*^/C^−^)=+1.76 V vs SCE) of thioxanthylium photoredox organocatalyst (TXT). Under green LED irradiation, diverse cyclobutane products **48** were achieved in good yields and high stereo‐ and regioselectivity as described in Scheme 26.

**Scheme 27:**
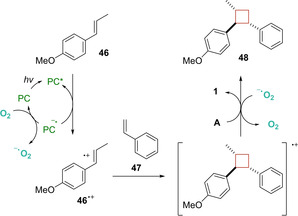
Proposed SET mechanism of the reaction.

According to Stern‐Volmer experiments, the excited organocatalyst should oxidize the electron‐rich styrene **46** via SET leading to the corresponding radical cation intermediate **46^⋅+^**. At this point, this latter undergoes [2+2] cross‐cycloaddition with **47** and, after a SET with the superoxide radical O_**2**_^−^, the desired product **48** is released (Scheme 27). Since the reaction does not proceed well under nitrogen atmosphere, the authors hypothesized that the oxygen could work as a key mediator.

**Scheme 28:**
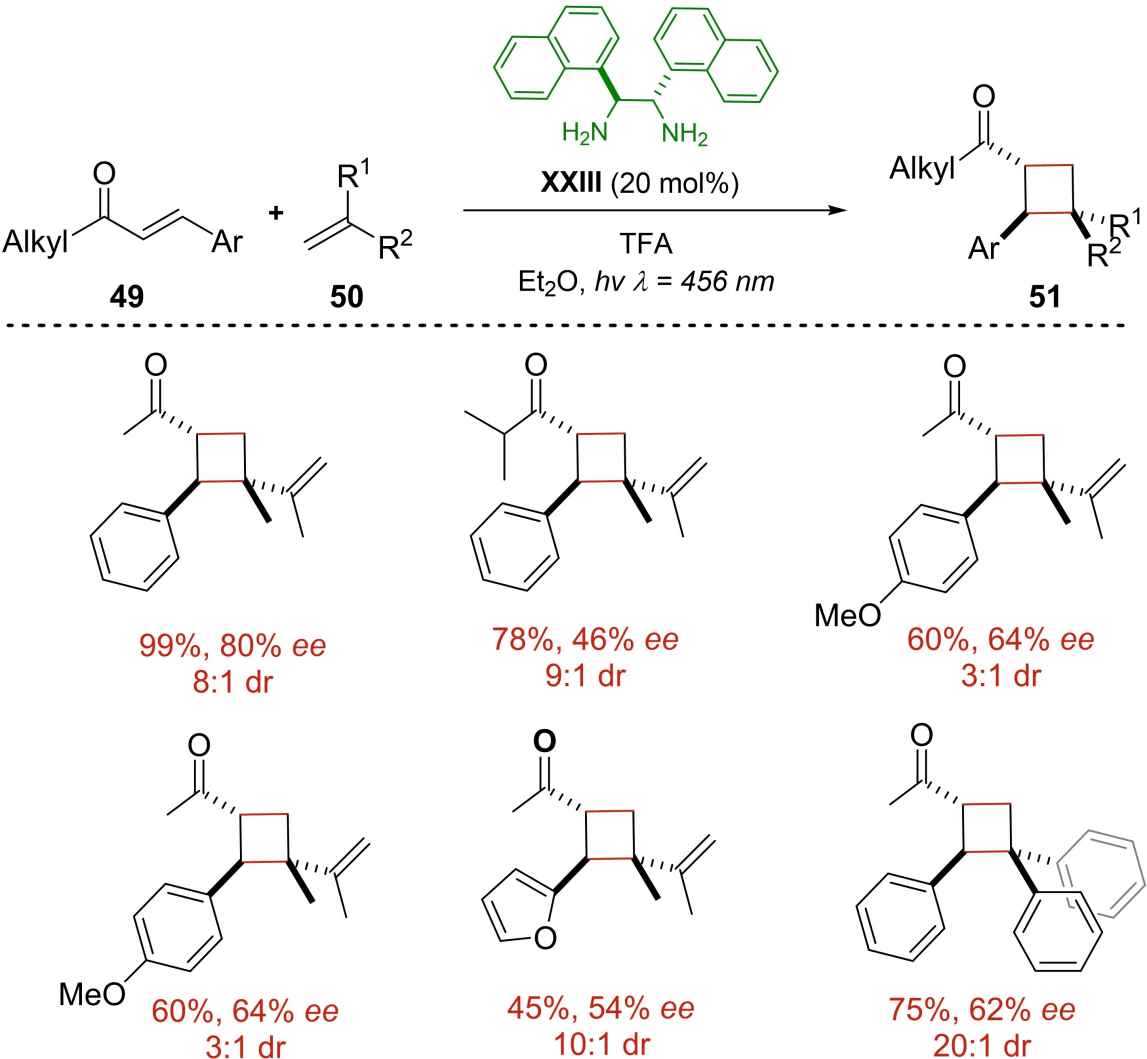
Intermolecular photocycloaddition of acyclic enones **49**.

In 2020, our group endeavored to contribute to the field and designed a protocol based on iminium ion catalysis which permits the synthesis of enantioenriched cyclobutanes starting from acyclic enones **49** (Scheme 28).^[41]^ The strategy steps on the generation of an intramolecular charge transfer complex between an electron‐rich moiety at the aminocatalyst **XXIII**, and the generated transient electron‐poor iminium ion. A wide set of compounds **51** was achieved in good yield and enantioselectivity, and several interesting observations during the study were addressed, mainly related to the proposal that a singlet excited state is the one that operates in the reaction instead of a more classical triplet state pathway.

**Scheme 29:**
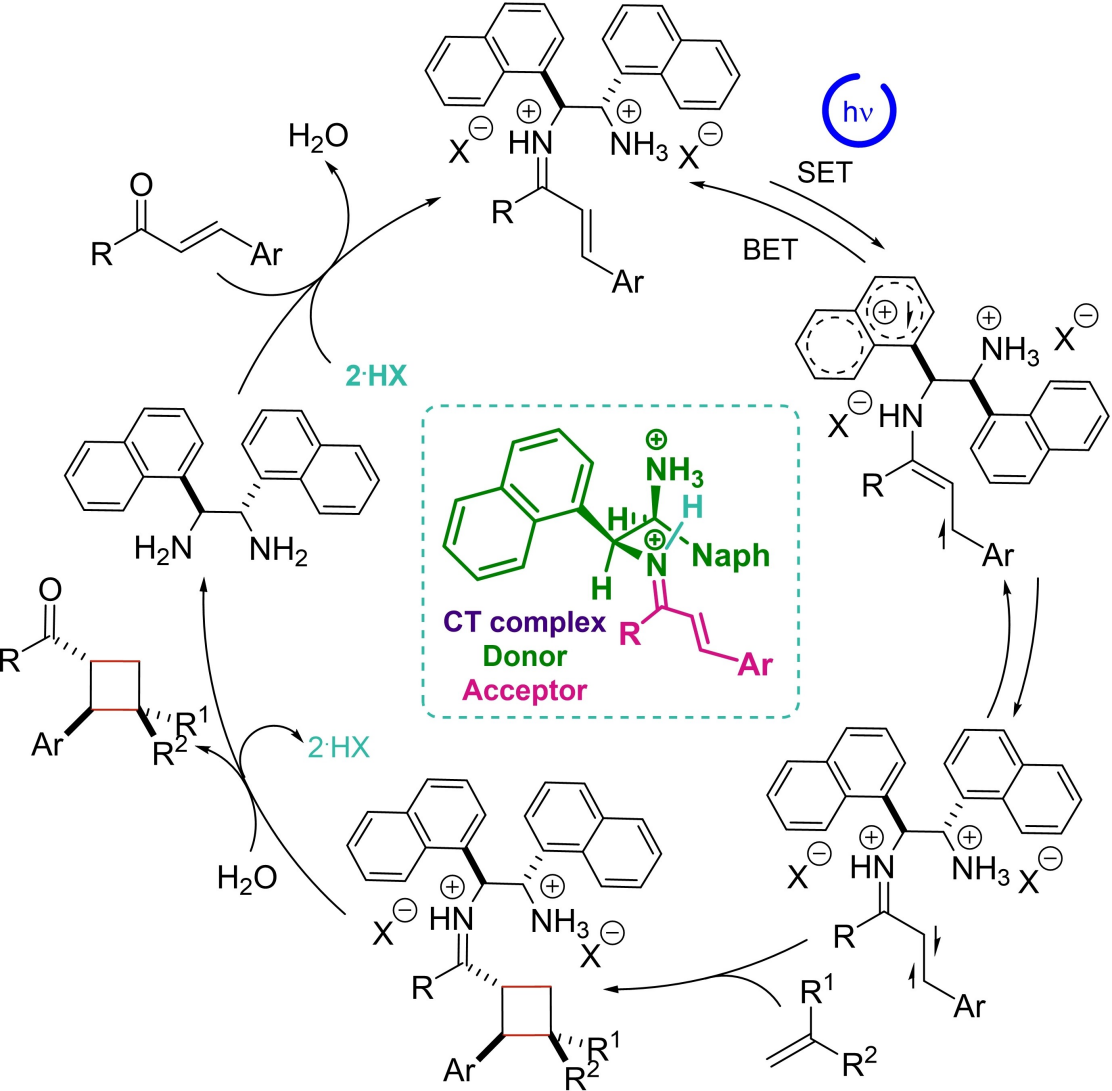
Charge‐transfer promoted [2+2] photocycloaddition.

Joined to control tests, DFT calculations helped to suggest a plausible catalytic cycle which is triggered by irradiation with blue LEDs of an iminium ion and consequently population of a singlet excited state (S_1_). Afterwards, this evolves reacting with the proper olefin **50** generating a biradical species that is sufficiently stable to afford the cyclobutane core, lastly releasing the diamine species **XXIII** in order to close the catalytic cycle (Scheme 29).

**Scheme 30:**
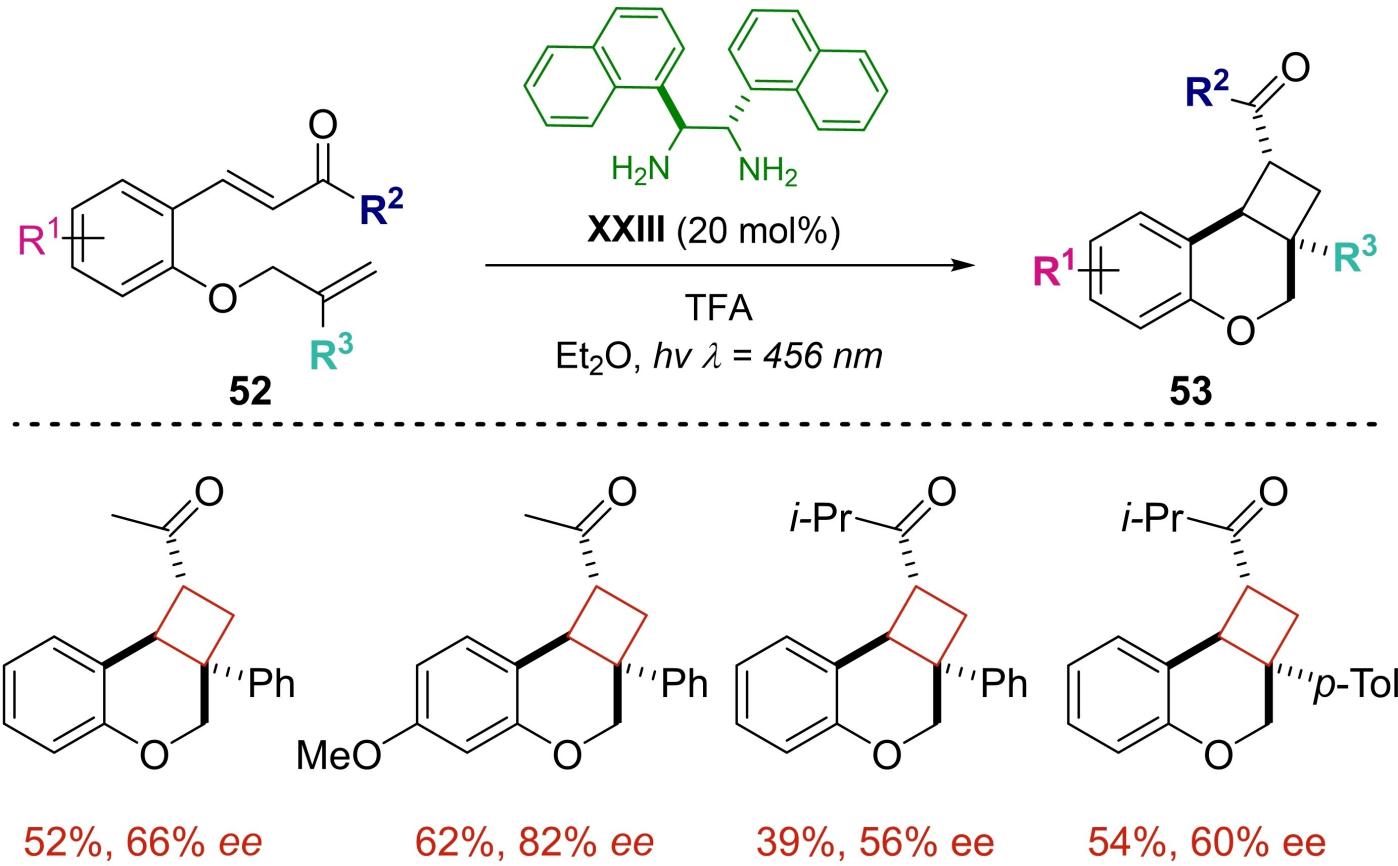
Intramolecular photocycloaddition of enones.

In the light of the satisfying results, the strategy was taken to the synthesis of enantioenriched tricyclic ethers **53**, proving its robustness, since this time an intramolecular photocycloaddition was the challenge to tackle.^[42]^ Smartly, enones **52** were used as starting materials and diamine **XXXIII** proved again to be the most suitable catalyst in the formation of the key charge‐transfer (CT) complex (Scheme 30). Following the designed protocol, several tricyclic ethers were obtained, displaying a wide tolerance in the substitution pattern, as the targeted compounds were isolated with good yield and enantioselectivity. Regarding the mechanism of the reaction, the photocatalytic cycle operates in an analogous version to the previous work, starting with the excitation of a chiral iminium ion intermediate, unshackling the corresponding cycloaddition steps.

Especially over other transformations, [2+2] photocycloadditions have been employed in the synthesis of natural products and interesting molecules from a biological standpoint. Namely, the group of Sarlah has extensively contributed to the field,^[43]^ by designing a general and trustable protocol for the dearomatization of arenes under visible light conditions.

## 3 Hetero‐photocycloadditions

**Scheme 31:**
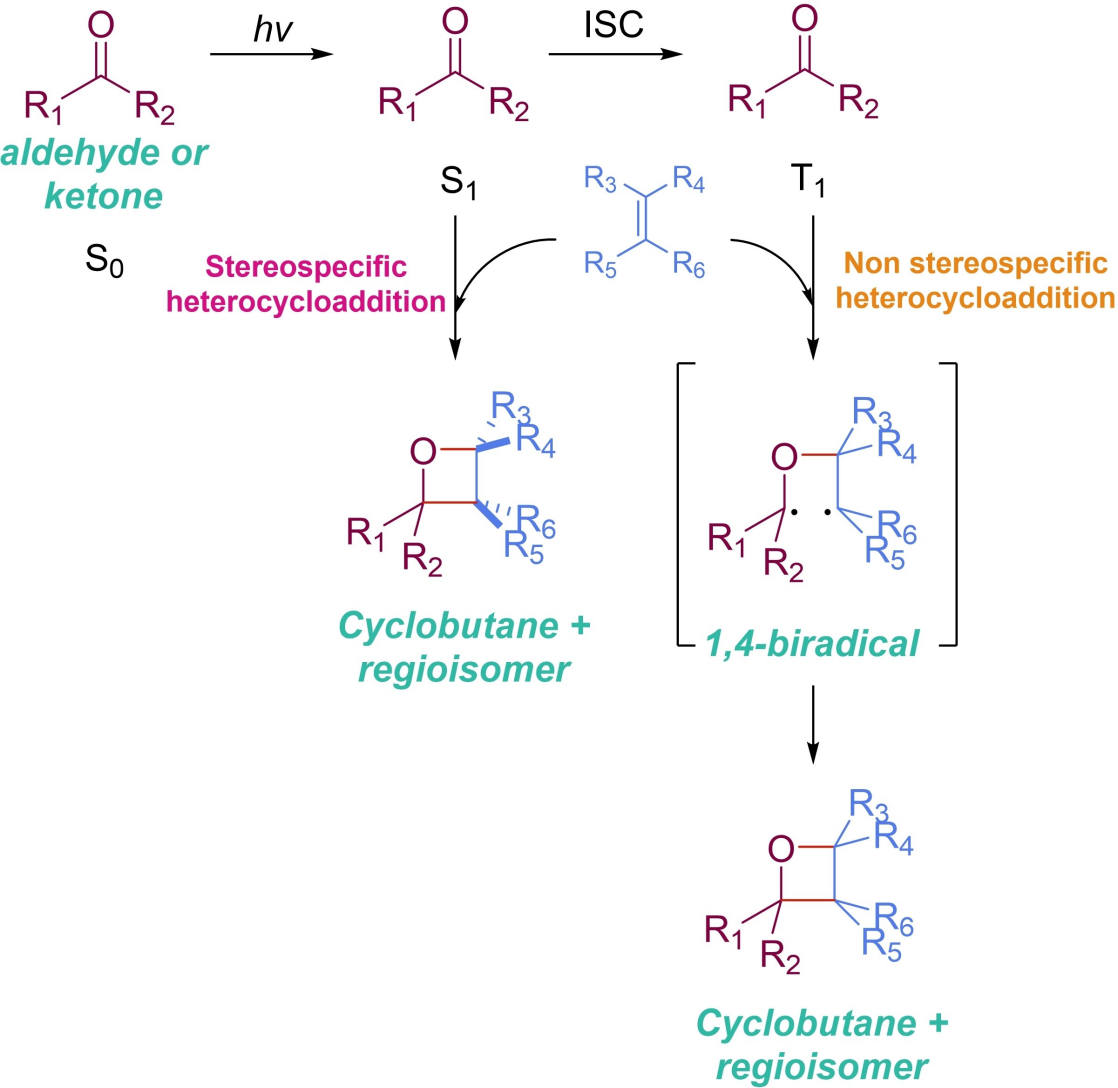
General overview of Paterno‐ Büchi reaction.

In 1954, swiss chemist George Büchi decided to reinvestigate Emauele Paternò’s foundings when exposing to sunlight (for 104 consecutive days!) a solution of benzaldehyde and 2‐methyl‐2‐butene: the formation of the corresponding oxetane as a mixture of isomers.^[44]^ Since then, the photochemical cycloaddition between a carbonyl system and an alkene to form oxetane derivatives is known as the Paterno–Büchi (PB) reaction. Loosely explained, the carbonyl substrate is excited upon irradiation to its singlet state (*S_1_*). The evolution of the overall transformation when departing from this latter excited state witnesses a stereospecific cycloaddition, where the stereochemical information of the alkene is translated to the final oxetane moiety. However, in the majority of cases, the *S_1_* evolves to the carbonyl triplet state (*T_1_*) via ISC. Addition to the olefin in matter gives place to a 1,4‐biradical intermediate and finally the most stable conformer collapses giving place to the target product, following a non‐stereospecific course (Scheme 31).^[45]^

**Scheme 32:**
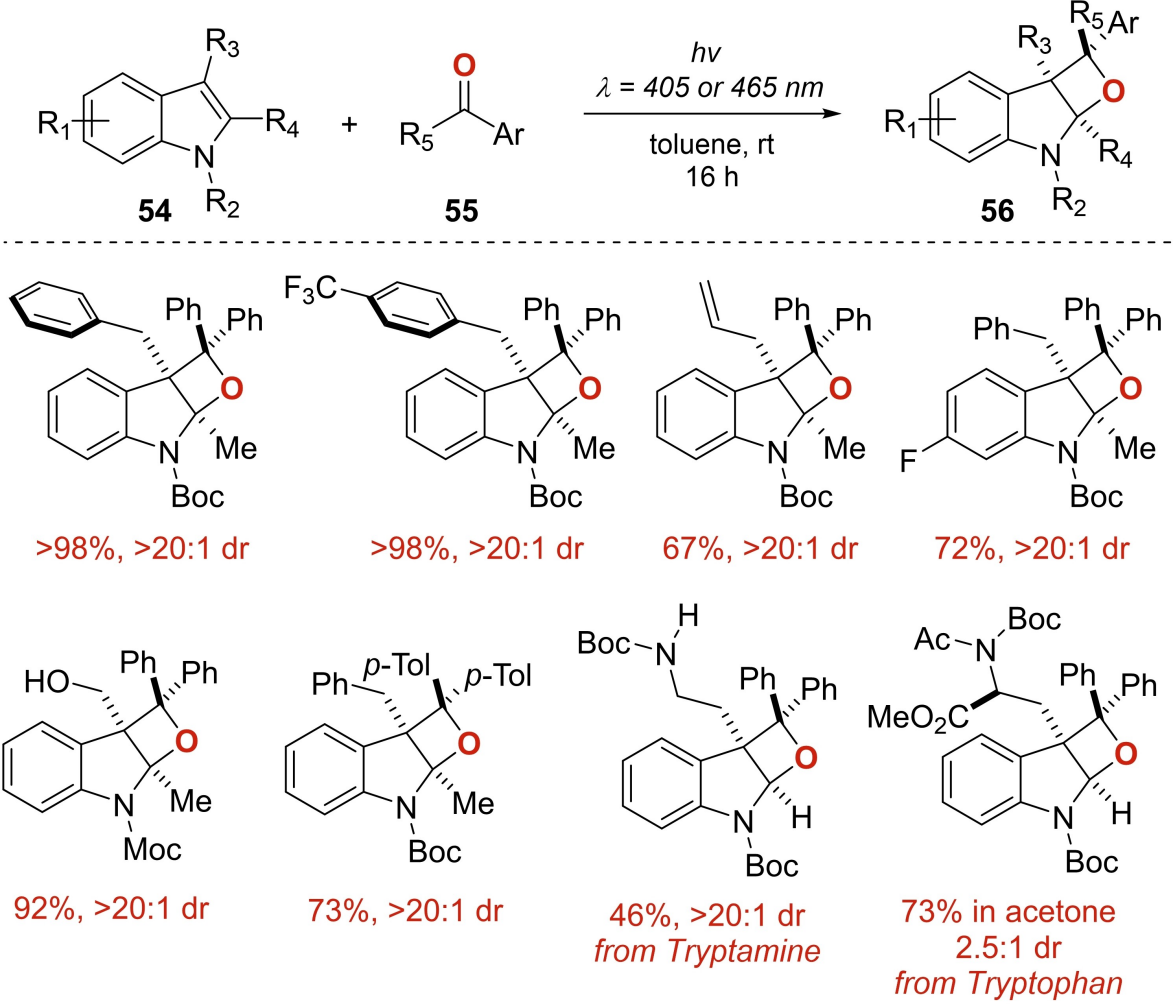
Synthesis of oxeto‐indolinic polycycles through a Paterno‐Büchi reaction.

In 2020, Companyó and Dell'Amico described the unprecedented use of visible light in the construction of oxeto‐indolinic polycycles **56** through a PB reaction, without the need of an external photocatalyst (Scheme 32).^[46]^ The work shines as a breakthrough in the literature, as it successfully addresses several of the most frequently encountered limitations on this fabled reaction: 1) the need of UV light irradiation, 2) low regio‐ and diastereo‐control, 3) the use of metallic photocatalysts. Joined to these facts, the authors were able to apply the designed methodology in gram scale by implementing a microfluidic photoreactor setup in the synthesis of bioactive compounds.

**Scheme 33:**
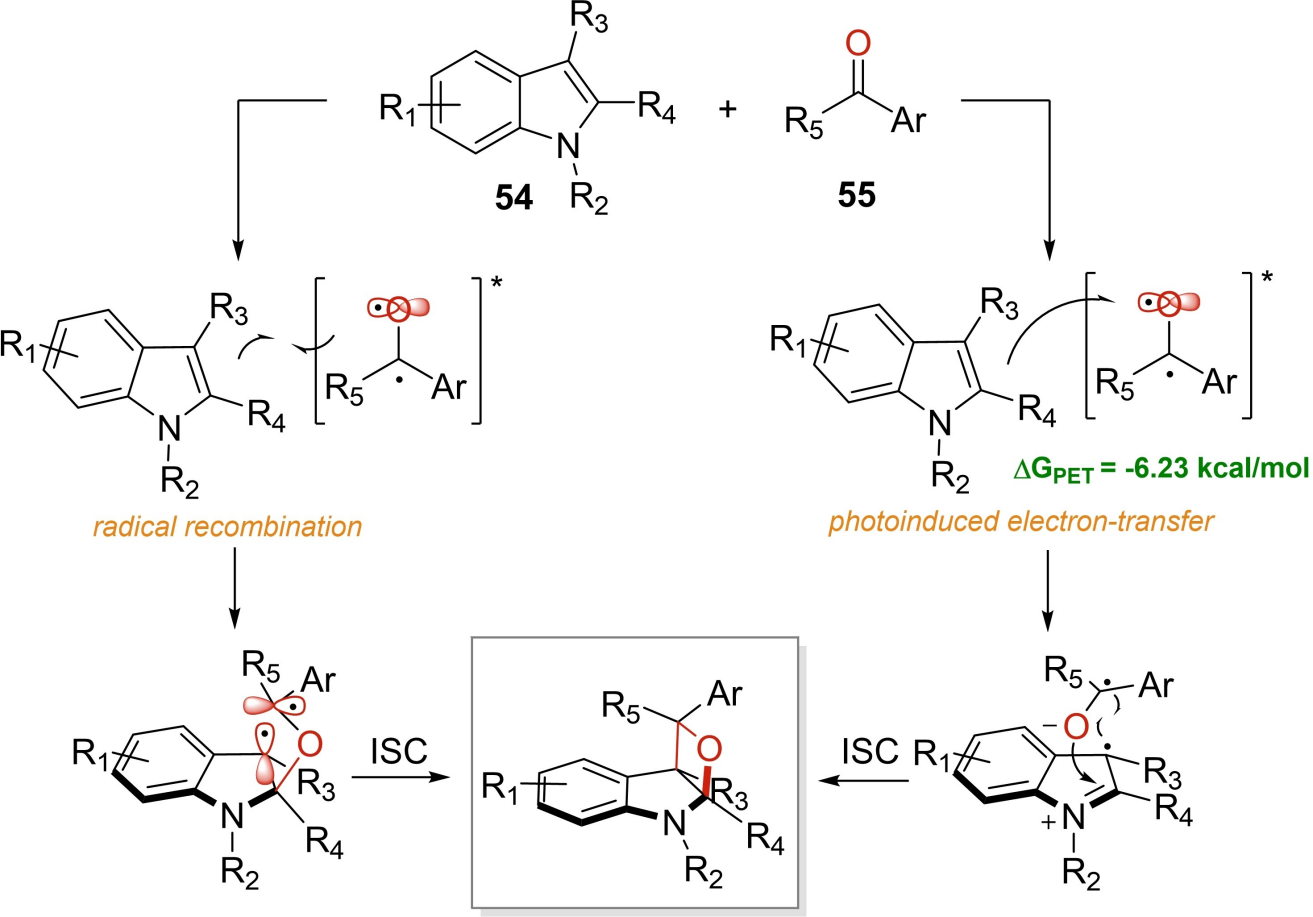
Proposed mechanism of Paterno‐ Büchi reaction.

Based on the results of stereocontrol and additional mechanistic tests, they were able to propose that two pathways might be feasible in the obtention of the final products **56** (Scheme 33). As named in the principal article, path A suggests a classic radical combination between the indole **54** and the excited triplet state T_1_ of the ketone **55**, and after final ISC the product **56** is obtained. This proposal follows a non‐stereospecific course (as mentioned previously, Scheme 31 *vide supra*), therefore, regio‐ and stereocontrol should be steered by steric factors. Alternatively, path B invokes a photoinduced electron transfer (PET) from the indole **54** to the excited ketone **55**. This proposal matches perfectly with the high stereoselectivity because either PET or S_1_ trapping, are faster processes compared to T_1_ radical combination and no time for molecular reorganization is available.

**Scheme 34:**
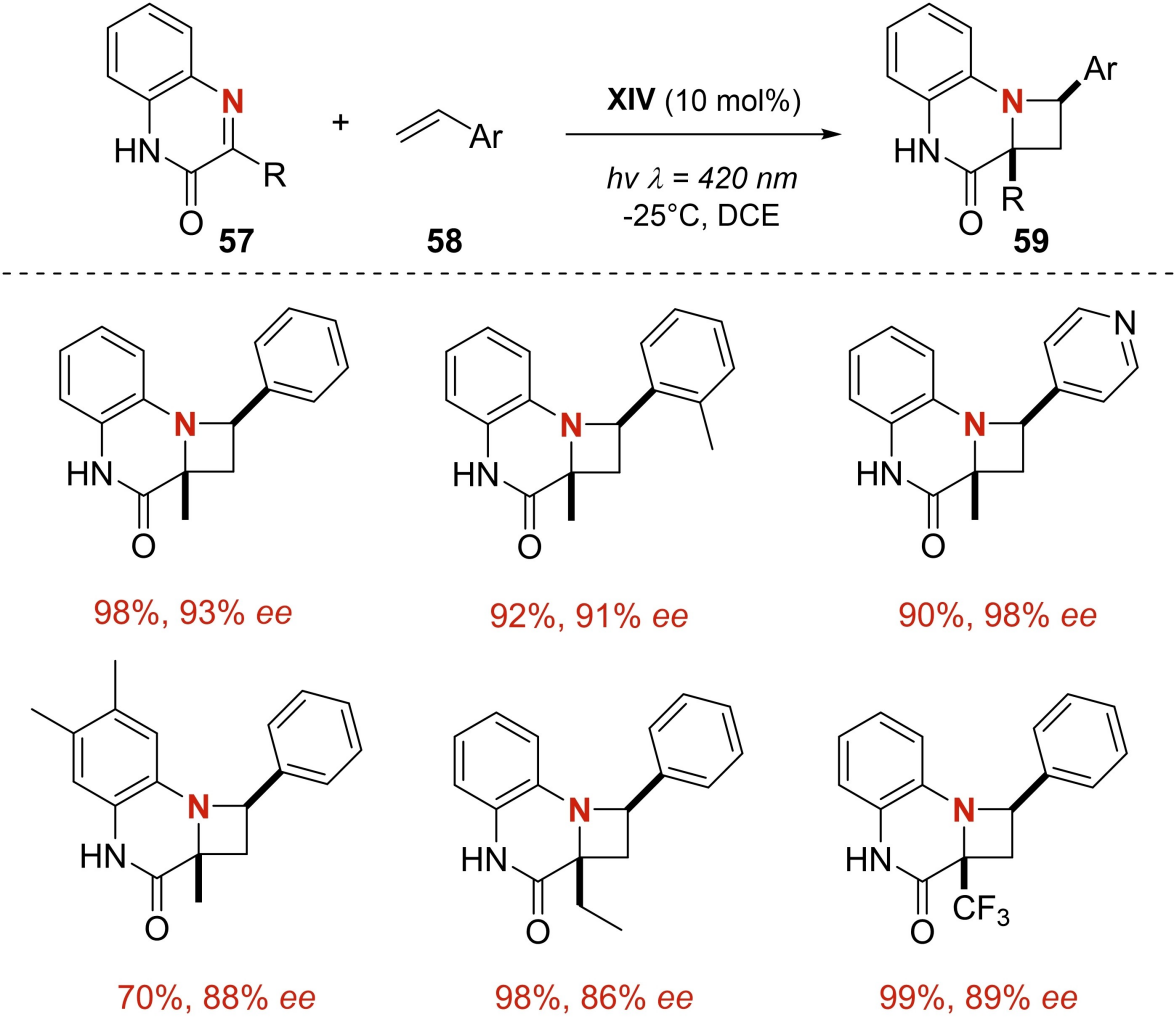
Synthesis of azetidines through aza‐Paterno‐ Büchi reaction.

Counterintuitive but true, [2+2] photocycloaddition between a C=N moiety and a suitable olefin (known as the aza‐Paternò Büchi reaction) remains scarcely explored. This gap in the literature is not due to an unwillingness from the scientific community, but to the inherent difficulty to design a predictable protocol. It has been well documented that the imine bond is susceptible to uncontrolled isomerization, rearrangement, fragmentation, and hydrolysis. Despite these challenging issues, brilliant methodologies have achieved the craved azetidine core, namely by Sivaguru^[47]^ and Schindler.^[48]^ However, the use of metallic photocatalysts or stoichiometric amounts of the chiral substrate, remain as a still‐to‐tackle limitation. In this regard, the group of Bach reasoned that a key strategy to successfully achieve the aza‐PB reaction, is by means of triplet sensitization. In accordance with their experience in the use of bifunctional catalyst **XIV** as triplet sensitizer under irradiation at 420 nm, the research group crafted an interesting protocol which leads to the named azetidines **59** in an enantioselective manner starting from quinoxalinones **57** and various styrene derivatives (Scheme 34).^[49]^

**Scheme 35:**
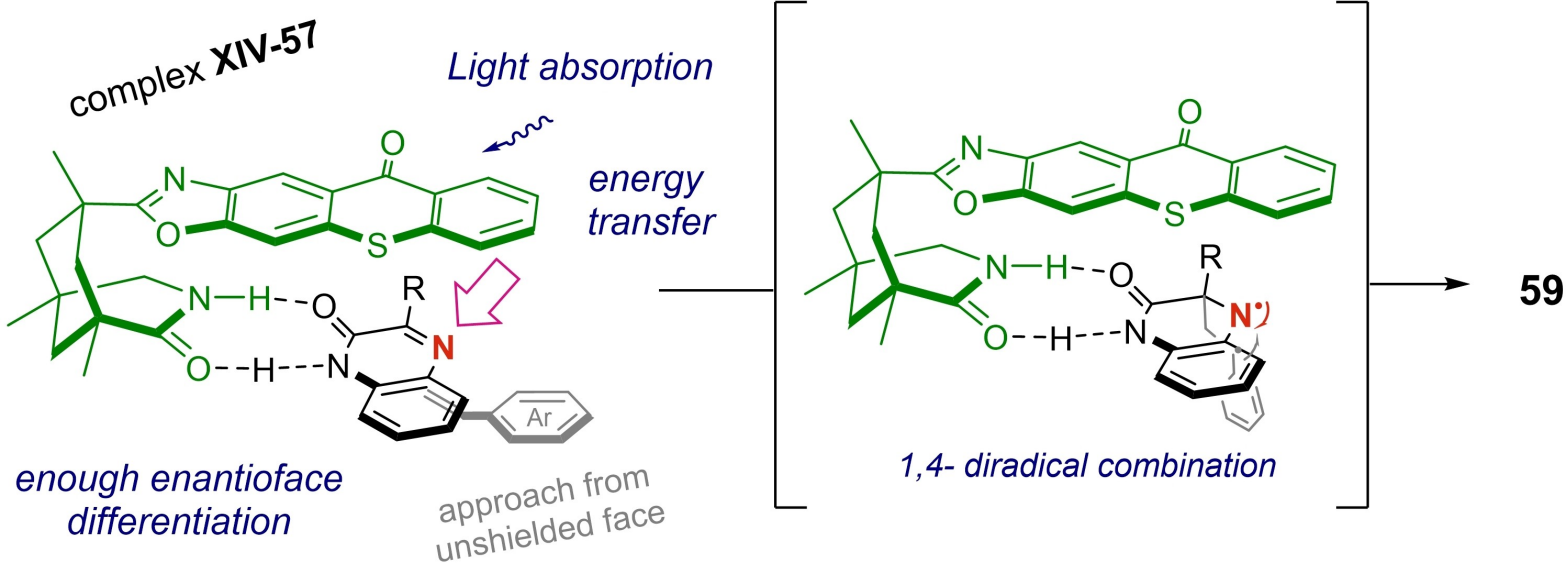
Mechanistic model for sensitization and chiral control of aza‐Paterno‐ Büchi reaction.

Taking into account previous studies on catalyst **XIV**, the authors suggest that hydrogen bonding to quinoxalinones **57** enables a triplet energy transfer and later the construction of a C−C bond formation within complex **XIV** **57**. Having preferentially shielded the *Si* face of **57**, interaction with the corresponding styrene **58** occurs in the *Re* face. The resulting 1,4‐diradical collapses with defined diastereoselectivity into the final azetidine **59** (Scheme 35).

**Scheme 36:**
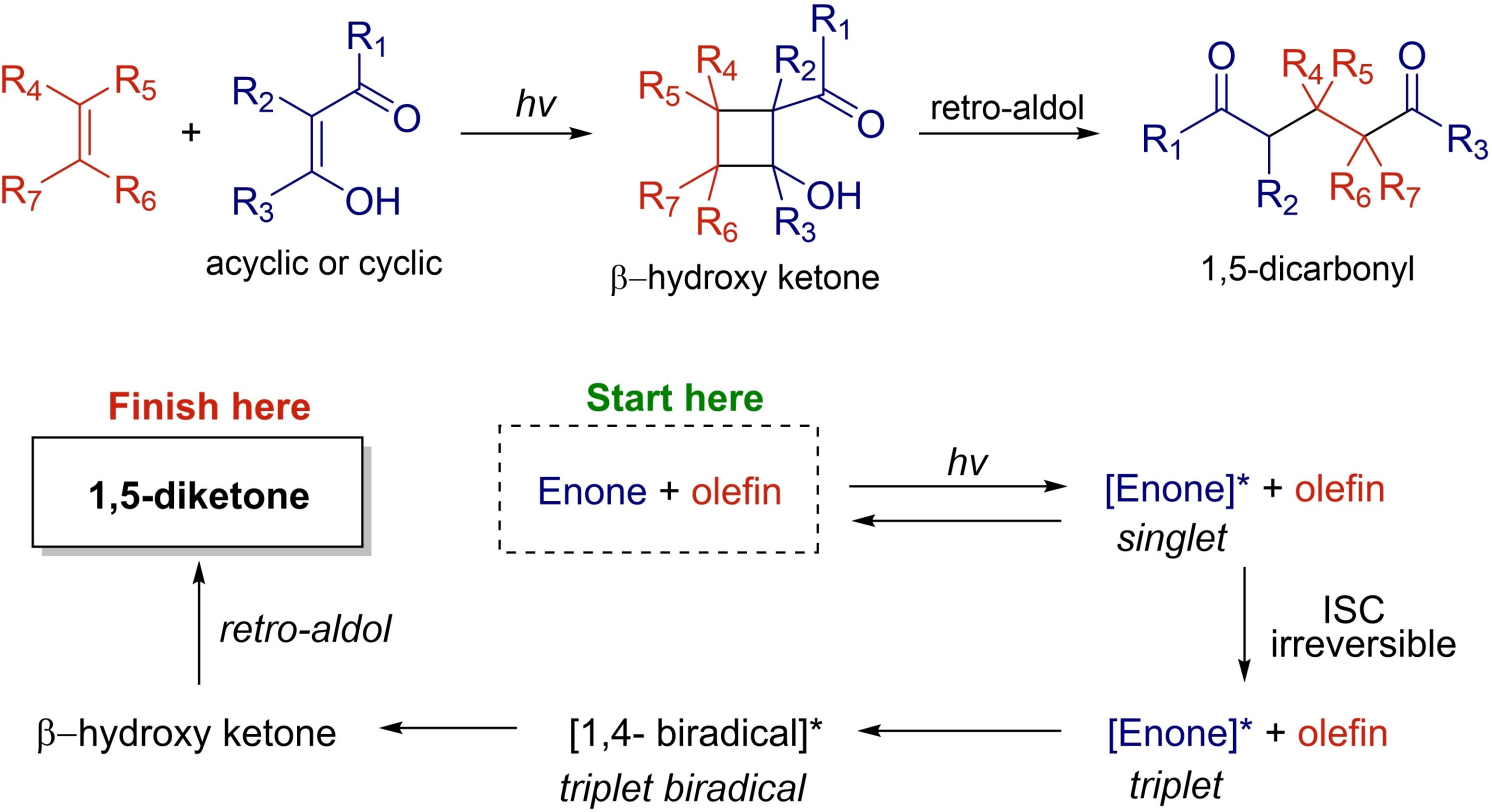
General overview of de Mayo reaction.

Another anecdotal milestone was made in the early 1960’s, when Paul José de Mayo described that irradiation of 1,3‐diketones (acetylacetone was used in his first paper) in the presence of another olefin, led to 1,5‐diketones.^[50]^ Later, it was identified that the actual cycloadduct was the enolized form of the 1,3‐diketone (the corresponding β‐hydroxy ketone), enabling the [2+2] photocycloaddition. In the last stage of the transformation, the resulting cyclobutanol derivatives, undergo spontaneous retro‐aldol condensation, giving place to 1,5‐diketones (Scheme 36).

Unsurprisingly, several methodologies have been developed since then, and the utility of the reaction has been proved in the total synthesis of interesting natural products as well.^[51]^ However, as far as we concern, only one example which employs visible light without the need of a metallic photocatalyst is currently available in the literature.

**Scheme 37:**
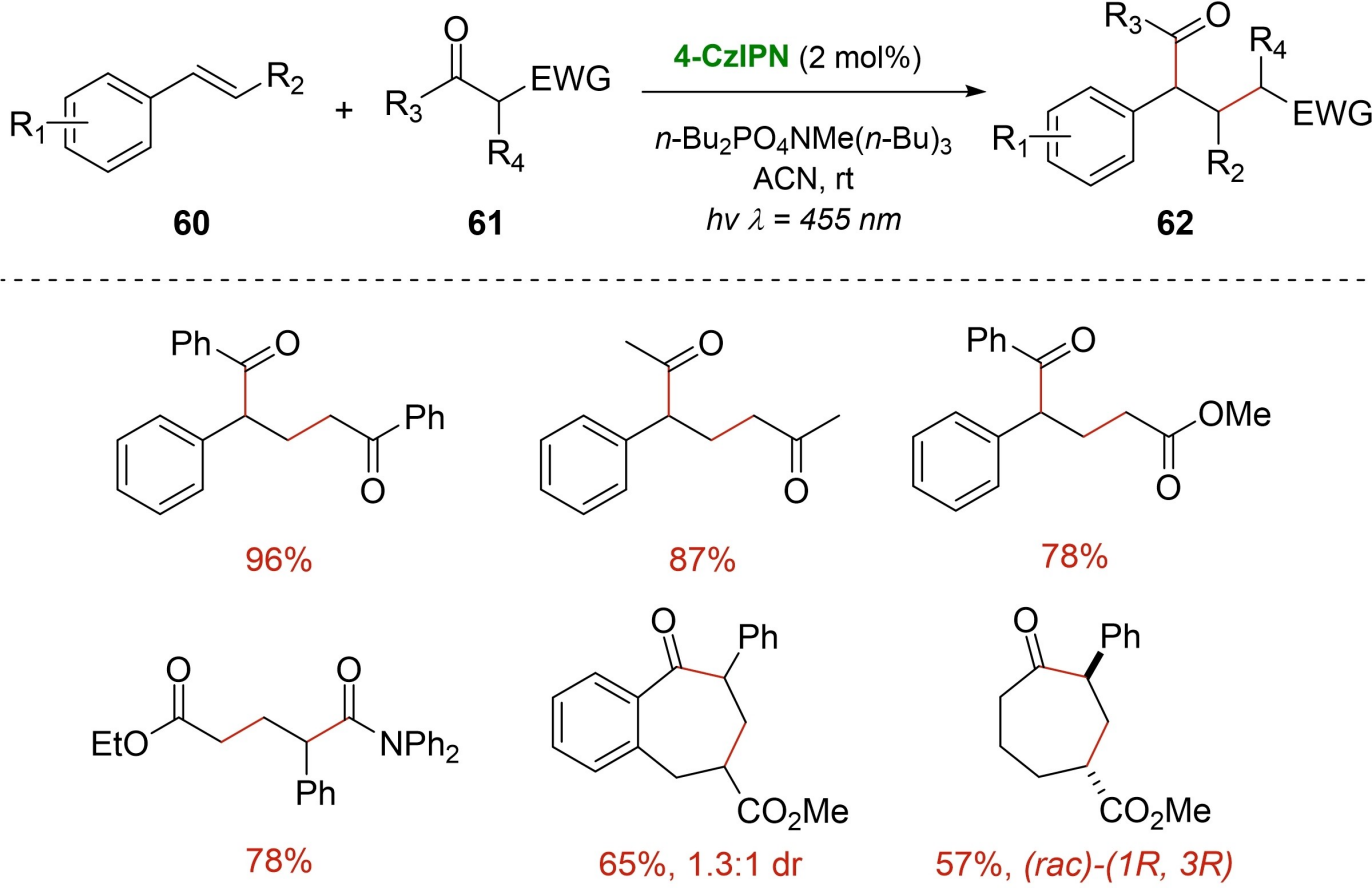
4‐CzlPN‐catalyzed de Mayo reaction.

In 2018, Marzo, König and coworker, described the use of organic photocatalyst 4CzIPN as photosensitizer when aiming to synthesize 1,5‐dicarbonyls **62**. Their investigations allowed them to carry out the de Mayo reaction in satisfactory yielding, starting from conventional 1,3‐diketones **61** and various styrene derivatives **60** using acetonitrile as solvent and blue light as energy source (Scheme 37).^[52]^

**Scheme 38:**
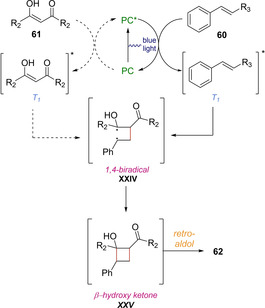
Proposed mechanism of the 4‐CzlPN‐catalyzed de Mayo reaction.

Based on experimental observations, a detailed analysis of redox potentials and previous reports, the authors suggest that a photocycloaddition pathway promoted by photosensitization is more likely to operate, rather than the direct oxidation of the β‐diketone (Scheme 38). Therefore, the proposal starts with the excitation of the photocatalyst, followed by energy transfer to either the olefin **60** or diketone **61**, populating its corresponding triplet excited state. Later on, these excited species might react with their corresponding ground state partners, leading to a common 1,4‐biradical intermediate **XXIV**, adopting the most prefer conformation. From this radical combination, cyclobutanol **XXV** is generated, followed by the typical retro aldol condensation. As stated by the authors, according to laser flash photolysis, it would be difficult to elucidate whether the energy transfer to styrene **60** or 1,3‐diketone **61** would be predominant, however, several indirect evidence allowed them to opt for direct photosensitization of **60** (styrene) as the predominant pathway.

## 4 [4+2] Cycloaddition. Crafting Six Membered Rings

In 1927, Otto Diels and Kurt Alder, untangled the misassigned structure of the cycloadduct between *p*‐quinone and cyclopentadiene.^[53]^ This [4+2] cycloaddition constitutes one of the most powerful carbon‐carbon bond forming reaction for the synthesis of diverse six‐member rings.^[54]^ However, high efficient reactivities and yields are ensured only involving electron‐rich dienes and electron‐poor dienophiles partners as well‐explained by the frontier molecular orbital.^[55]^ Therefore, strict and environmental unfriendly conditions as high temperatures and strong acids are required when electronically mismatching dienes/dienophiles reaction couple are used, strongly constraining their application in chemical and pharmaceutical industries. In many examples, electronically mismatching [4+2] cycloadditions were promoted by electron oxidation in presence of stoichiometric oxidant species (for example using iminium salts) or by photoinduced electron transfer (PET) with photosensitizers, via the generation of radical cation intermediates derived from electron‐rich dienophiles.[^56^, ^57^] Furthermore, to overcome these limitations, visible light‐mediated Diels Alder reactions via open‐shell radical cation or neutral intermediates provide attractive and sustainable alternative approaches avoiding the use of any stoichiometric oxidant or high energy radiations.

In this context, Yoon, Zhang, Arai and Ohkuma groups have contributed to the field by describing diverse visible‐light‐promoted Diels Alder and aza‐Diels‐Alder reactions, catalyzed by Ru and Cr complexes.^[58]^ These reports offer excellent reactivity towards electron‐rich dienophiles through radical cation intermediates. However, in all cases, transition metal based photocatalyst were employed. Based on our interest in metal‐free catalysis, our purpose is to summarize the more recent visible‐light [4+2] methodologies catalyzed by commercially available, cheaper, and non‐toxic organic photocatalysts.

**Scheme 39:**
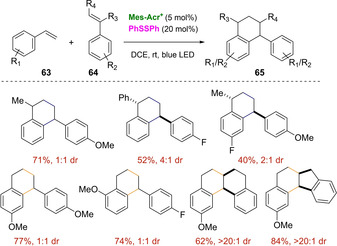
Mes‐Acr^+^‐catalyzed [4+2] photocycloaddition.

In 2017, Nicewicz, Huang and co‐workers reported the light‐mediated synthesis of tetralin derivatives **65** starting from a pair of styrene molecules.^[59]^ Several particularities of the protocol turn this procedure into a remarkable achievement for [4+2] cycloadditions. For instance, the inherent possibility to undergo [2+2] pathway is suppressed by involving a disulfide as cocatalyst in non‐polar media, achieving high levels of chemoselectivity. Continuing with the troubleshooting of the protocol, homodimerization during the craved Diels‐Alder cycloaddition was circumvented by carefully designing the pair of starting styrenes, which required to have different electronic and steric properties one from each other. The overall chemo‐ and regioselectivity of the transformation allows to obtain a sole family of products among the wide list of possibilities, and a key factor to achieve this, was the slow addition of the styrene swifter to get oxidized over the more nucleophilic one. Noteworthy, under the optimal reaction conditions, tricyclic and tetracyclic tetralin derivatives **65** were smoothly prepared in high yields and as single diastereomers starting from indene and tetrahydronaphthalene as dienophiles (Scheme 39).

**Scheme 40:**
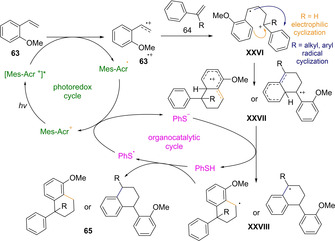
Proposed mechanism of the Mes‐Acr^+^‐catalyzed [4+2] photocycloaddition.

Based on preliminary isotope‐labeling experiments and previous studies on the hydrofunctionalization of styrenes, a polar radical crossover cycloaddition (PCCR) reaction mechanism was suggested (Scheme 40). Under blue LED irradiation, the excited state of acridinium catalyst [*E* ^*^_red_ (Mes‐Acr^+^/Mes‐Acr)=+2.18 V] oxidizes the electron rich styrene **63** promoting the [4+2] cycloaddition with the diene **64**. The resulting radical cation intermediate **XXVI** can undergo two possible cyclization pathways: electrophilic or radical depending on its structural/electronic nature. The electrophilic cyclization is favored with non‐α‐substituted dienophiles, whereas the radical pathway operates with α‐substituted styrene in response to the higher stability of the cation center. Independently from which route is followed, in both cases, a rearomatization event conducts to the tetralin product **65**.

**Figure 1:**
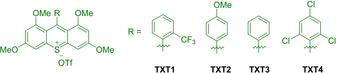
Structures of thioxanthylium‐based (TXT) salts.

In 2018, a family of thioxanthylium‐based (TXT) salts was introduced by Honda and Hoshino as new organophotoredox catalyst for metal‐free Diels‐Alder reactions (Figure 1).^[60]^

**Scheme 41:**
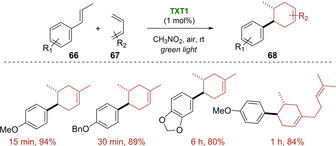
TXT1‐catalyzed [4+2] photocycloaddition

Choosing *trans*‐anethole and 2,3‐dimethylbutadiene as model reaction partners, these new **TXT1‐4** salts efficiently catalyzed the radical cation Diels‐Alder reaction under green light irradiation. This resulted in the preparation of the corresponding cyclohexene product with high yield in short reaction times (15–75 minutes). The best **TXT1** organophotocatalyst was also tested with different electron‐rich dienophiles **66** and dienes **67** such as isoprene and myrcene affording good results (Scheme 41). The utility of the system was further demonstrated on the gram scale setup, obtaining the desired product **68** in 84 % yield after 5 minutes of sunlight exposure. This latter proved unequivocally the ability of these photocatalysts to absorb light in the visible region (∼400–600 nm), offering high excited‐state reduction potentials (*E*_0_’(C^*^/C^−^)=+1.79−1.94 V vs SCE).

**Scheme 42:**
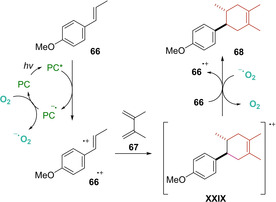
Proposed mechanism of TXT1‐catalyzed [4+2] photocycloaddition.

Joined to these observations, Stern‐Volmer experiments allowed the authors to suggest that an electron transfer event from *trans*‐anethole (*E*_0_’(C^*^/C^−^)=+1.24 V vs SCE) to the catalyst should occur smoothly, leading to the radical cation **66^+^**. This species undergo a [4+2] cycloaddition with **67** to generate the radical cation intermediate **XXIX**. Finally, a SET event from the superoxide radical O_2_^−^ (formed via reduction of molecular oxygen with the reduced photocatalyst) or *trans*‐anethole generates the desired product **68** (Scheme 42). However, according to quantum yield experiment, a radical chain process is favored.

**Scheme 43:**
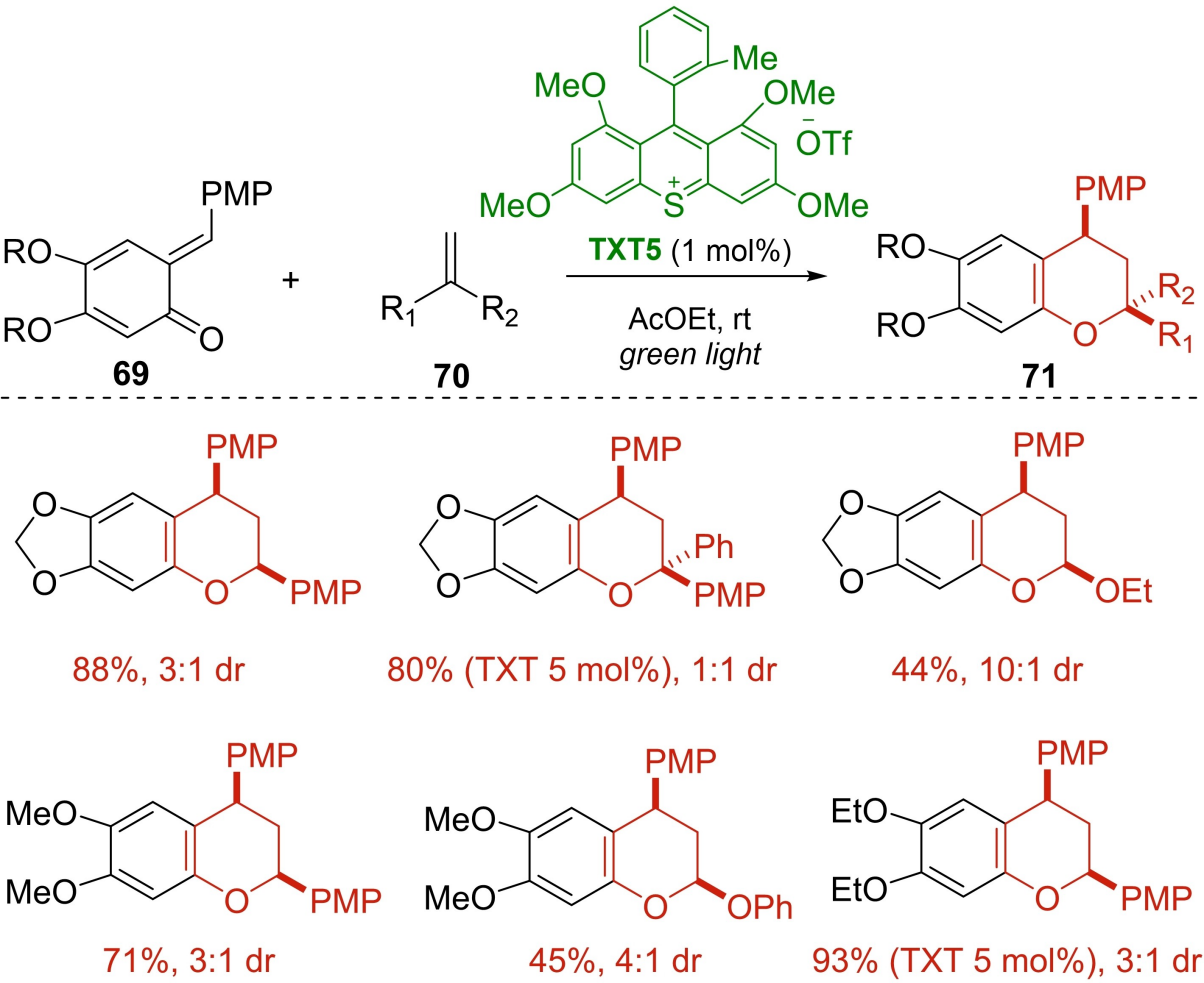
TXT5‐catalyzed [4+2] photocycloaddition of ortho‐quinone methides **69**.

**Scheme 44:**
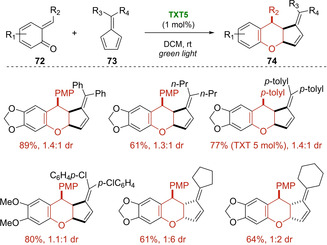
TXT5‐catalyzed [4+2] photocycloaddition of ortho‐quinone methides **72**.

It is noteworthy that the authors were able to extend the general idea to ortho‐quinone methides **69** and **72** developing mild and atom‐economical thioxanthylium‐catalyzed oxa‐Diels‐Alder reactions under green light irradiation (Scheme 43 and Scheme 44).[^61^, ^62^] In particular, several benzopyran derivatives **71** and **74** were smoothly synthesized in good to high yields and moderate diastereoselectivity using electron‐rich styrenes **70** and pentafulvenes **73** bearing aromatic and aliphatic groups as dienophiles. In both cases, aprotic polar solvents provided the best results of the oxa‐[4+2]‐cycloadditions possibly due to a better stabilization of the radical cation intermediates in this reaction media. A possible mechanism similar to that previously reported for *trans*‐anethole **68** was suggested. However, based on the quantum yield measurements (φ=0.21 and 0.15 respectively with styrenes and pentafulvenes), both oxa‐cycloaddition reactions should mainly proceed via photocatalytic pathways.

**Scheme 45:**
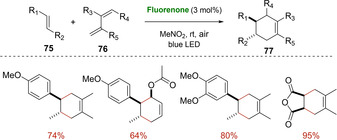
Fluorenone‐catalyzed [4+2] photocycloaddition of **75**.

**Scheme 46:**
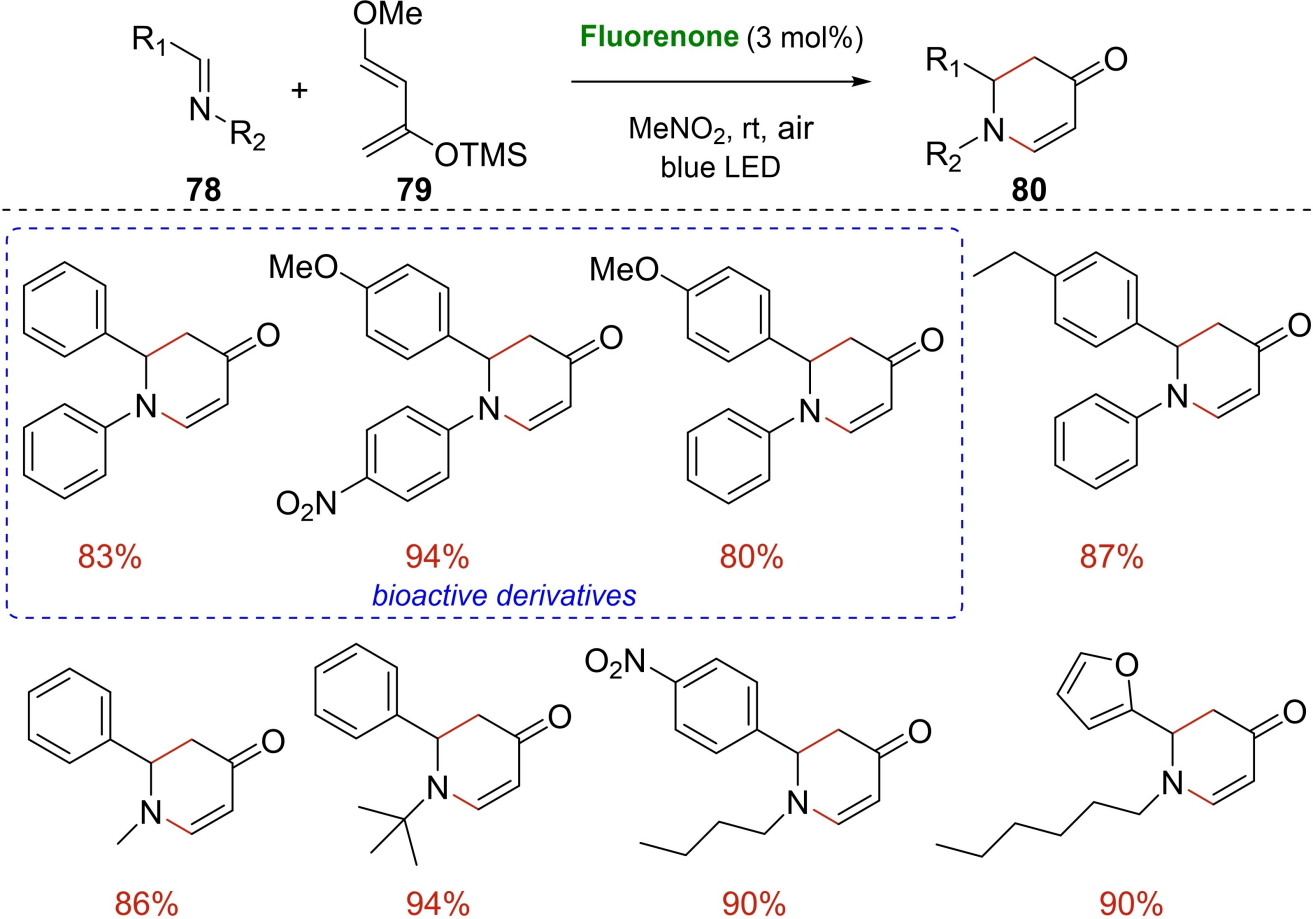
Fluorenone‐catalyzed aza‐[4+2] photocycloaddition of **78**.

In 2019, Das and co‐workers satisfactorily employed 9‐fluorenone as simple catalyst for metal‐free visible‐light mediated Diels‐Alder and Aza‐Diels‐Alder reactions (Scheme 45 and Scheme 46).^[63]^ This cheap and commercially available catalyst showed excellent reactivity with electron‐rich dienophiles **75** and **78** as *trans*‐anethole, 2,5‐furandione, *N*‐ethylmaleimide, smoothly leading to diverse cyclohexene products (**77** and **80**). Along with these latter, three different bioactive molecules already evaluated in vitro against a library of virus were prepared. The ketone‐catalyzed‐aza‐[4+2] cycloaddition involving imines was a useful strategy to achieve heterocycles in a single step and in an atom‐economical way. Control experiments along with mechanistic investigations revealed a possible chain propagation mechanism, in which the oxygen in the reaction media act as a crucial electron mediator to further improve the yields. The suggested mechanism is similar to that reported in Scheme 42. Also in this work, crucial is the initial excitation of the ketone to its photoexcited state by irradiation of visible light in order to promote a SET with the dienophile species. The redox potential value of the 9‐fluorenone (+1.7 V vs SCE) is, for example, high enough to oxidize *trans*‐anethole (+1.1 V vs SCE) in its ground‐state.

**Scheme 47:**
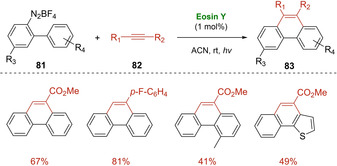
Eosin Y‐catalyzed [4+2] photocycloaddition of **81**.

In 2012, Zhou's research group reported an Eosin Y catalyzed [4+2] benzannulation of biaryldiazonium salts **81** with alkynes **82** under visible light irradiation.^[64]^ A variety of 9‐substituted or 9,10‐disubstituted phenanthrenes **83** were smoothly prepared with moderate to good yields (Scheme 47) via a cascade radical addition and cyclization sequence. The gradually addition of diazonium salts to the polar reaction mixture containing 3 equivalents of alkyne, provided the best results. Moreover, during the scope investigation, higher yields were achieved with more electron‐deficient alkynes probably attributed to their higher ability to undergo radical addition.

**Scheme 48:**
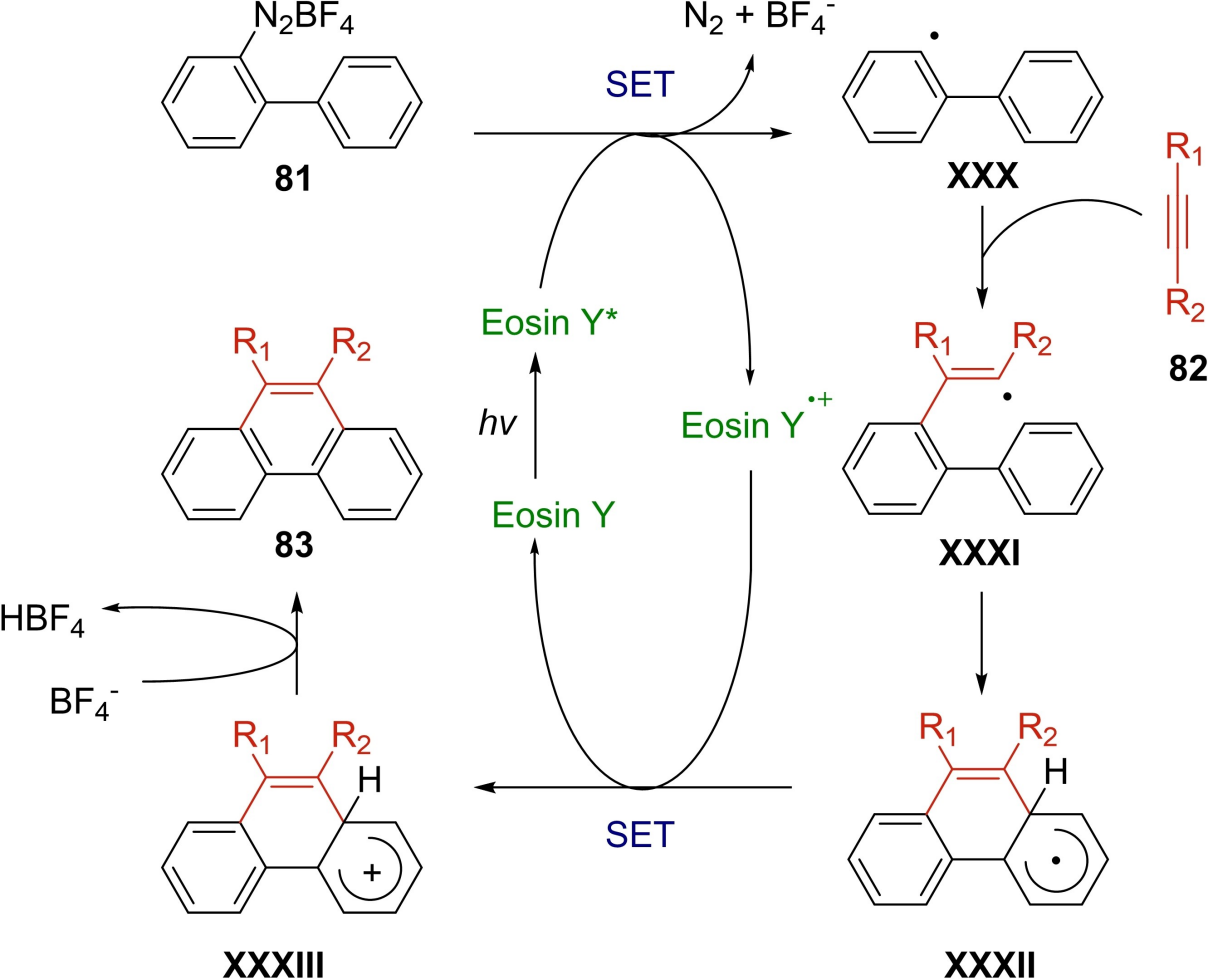
Proposed mechanism of Eosin Y‐catalyzed [4+2] photocycloaddition.

Indeed, the authors suggest a possible mechanism, in which the biaryl radical intermediate **XXX**, generated via SET with excited Eosin Y, interacts with the alkyne **82** leading to the vinyl radical **XXXI** (Scheme 48). Then, an intramolecular radical cyclization followed by a single‐electron oxidation of **XXXII** by Eosin Y^+^, should lead to the desired product **83**.

**Scheme 49:**
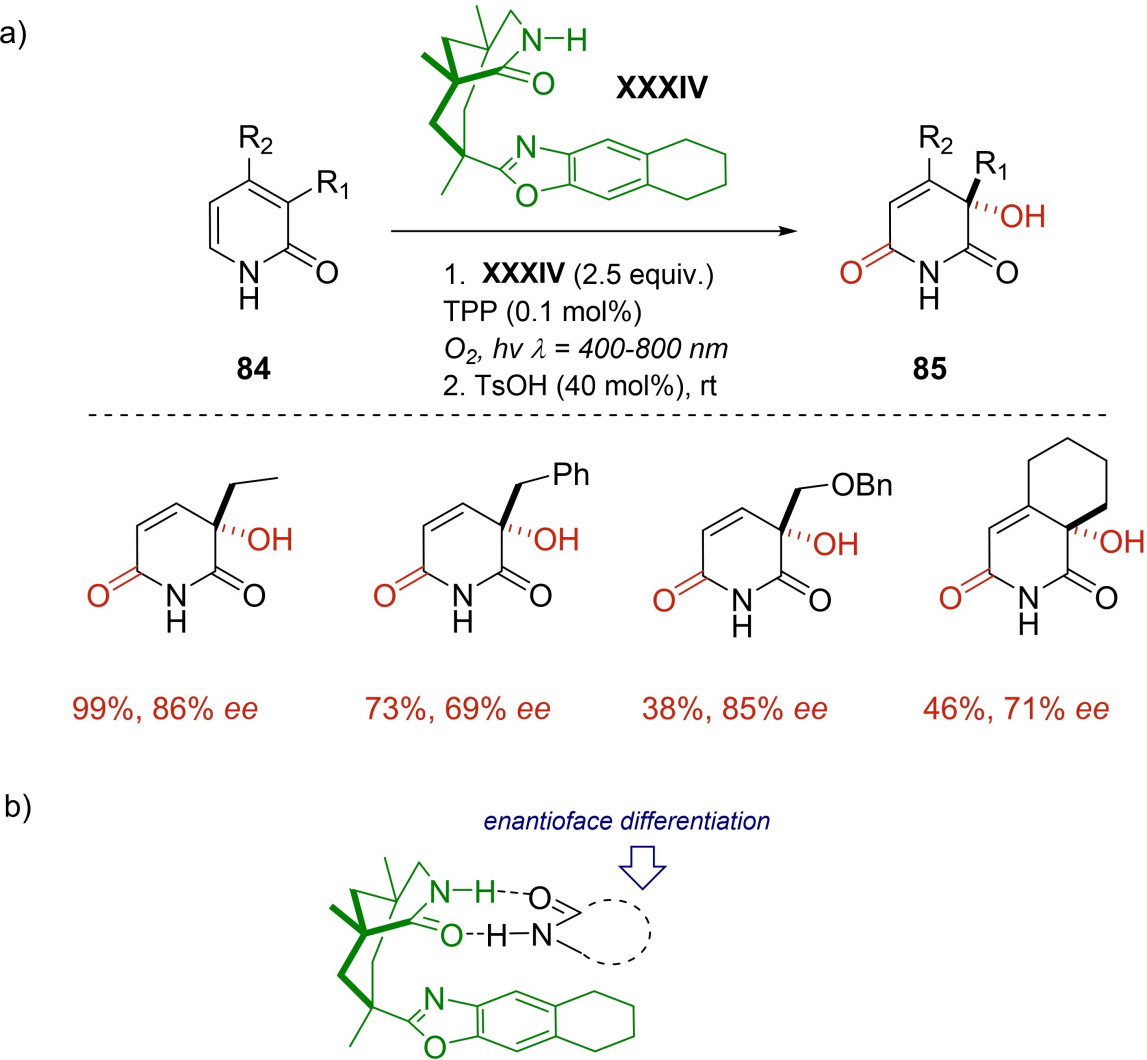
a) **XXXIV** as chiral organocatalyst for [4+2] photocycloaddition b) Mechanistic model for chiral control.

In the same year, Bach demonstrated that visible light irradiation of pyridones **84** in presence of chiral template **XXXIV** can successfully induce the enantioselective synthesis of 3‐hydroxypyridine‐2,6‐diones **85** via singlet oxygen [4+2] cycloaddition (type II photooxygenation) followed by Kornblum–DeLaMare rearrangement (Scheme 49a).^[65]^ This transformation is conducted under oxygen atmosphere at 400–800 nm using tetraphenylporphyrin (TPP) as external photosensitizer. An optimized protocol, consisting in three cycles of irradiation at low temperature (20 minutes) and acid treatment at room temperature (12 hours), was essential to minimize the preferred retro‐[4+2] cycloaddition, giving place to the products **85** in good yields. However, the acidic conditions showed to be deleterious in presence of ethers and benzylic groups leading to unavoidable side reactions and isolating the corresponding products in just moderate yields. At the same time, it is remarkable that all the final compounds were afforded with high levels of enantioselectivity (68–90 % ee) due to a good enantioface differentiation provided by the chiral template. The authors claim that the formation of 1 : 1 complex between the pyridone **84** and the template **XXXIV** stabilized by two hydrogen bonds should be involved, as depicted in Scheme 49b.

**Scheme 50:**
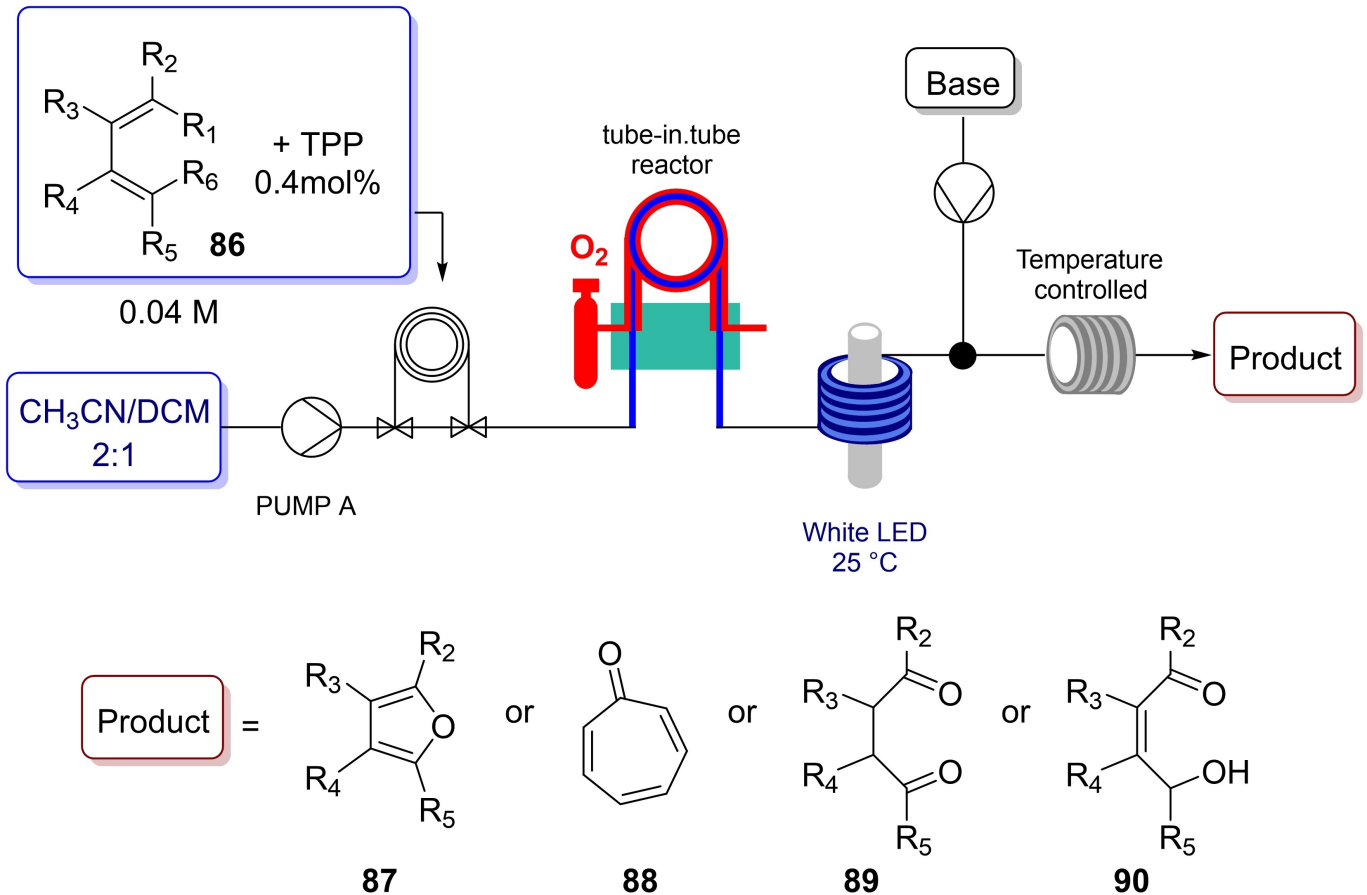
Continuous flow TPP‐catalyzed photocycloaddition.

In 2018, Oliveira and co‐workers developed the first example of continuous flow TPP‐catalyzed photooxidation of conjugated dienes **86** (Scheme 50).^[66]^ This end‐to‐end two steps setup (endoperoxidation and Kornblum–DeLaMare rearrangement) showed to be a convenient and reproducible approach for the preparation of diverse interesting compounds such as 1,4‐dicarbonyl building blocks **89**, substituted furans **87**, tropone **88** and hydroxyenones **90**. Additionally, the scalable version from 3 to 10 mmol was accomplished affording the product **90** in high overall yield.

**Scheme 51:**
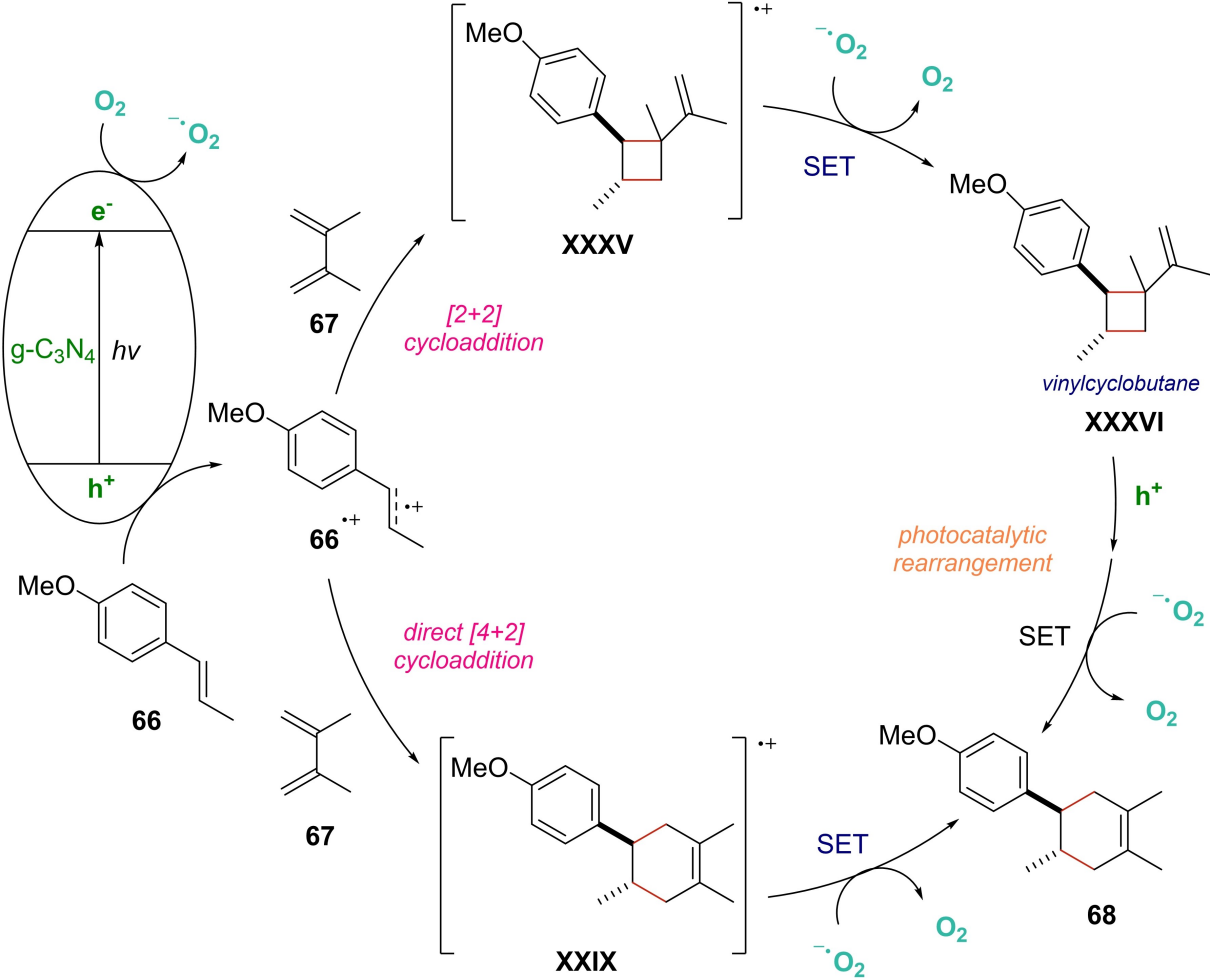
Proposed mechanism of g‐C_3_N_4_‐catalyzed heterogeneous photocycloaddition.

In 2017, Zhao and Antonietti reported a Diels‐Alder reaction with electron‐rich olefins **66** catalyzed by graphitic carbon nitride (g‐C_3_N_4_) as heterogeneous photocatalyst under visible‐light‐irradiation (Scheme 51).^[67]^ This protocol exhibits a large substrate scope, which shines by the high yields and excellent stereoselectivity of the obtained products **68**. The presence of oxygen in the reaction media was crucial for the success of the transformation. As a matter of fact, the authors describe its critical role as an active electron mediator. Interestingly, by GC‐MS and NMR analysis, vinylcyclobutane **XXXVI** was identified as a reaction intermediate, thus, hinting that a combination of two mechanisms might be operating in the overall reaction. 1) A direct [4+2] cycloaddition and 2) a [2+2] cycloaddition in which the previously mentioned vinylcyclobutane **XXXVI** evolves into the product **68** through a photocatalytic rearrangement. As concerning the behavior of the heterogeneous photocatalyst, the visible light irradiation should induce a charge separation providing an oxidative valence band and a reductive conduction one. In detail, the conduction‐band electrons are able to reduce the oxygen producing superoxide radicals O_2_^−^, whereas the valence band hole oxidizes the electron‐rich dienophile **66** into the corresponding radical cation **66^+^**.

**Scheme 52:**
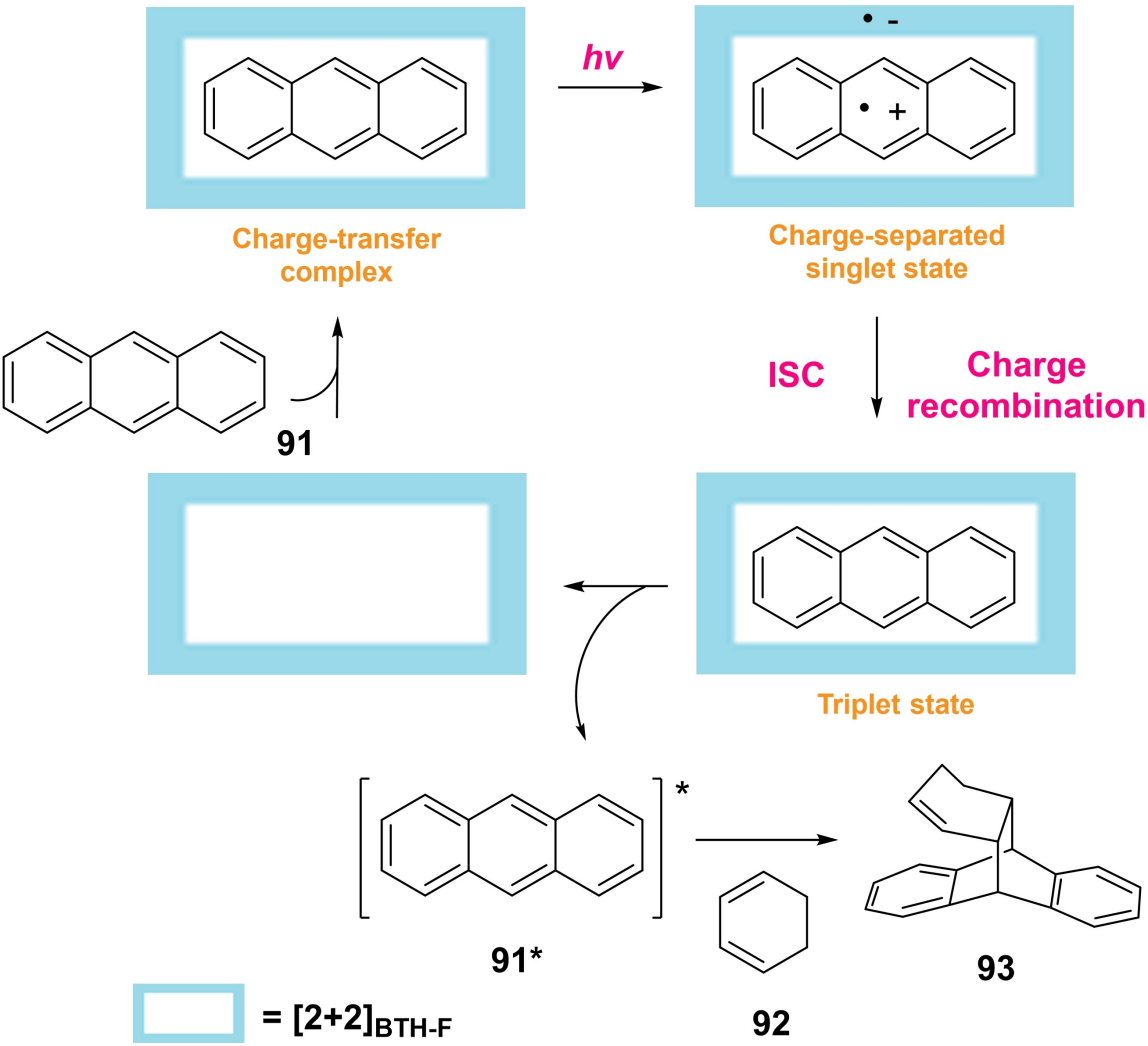
Proposed mechanism of supramolecular charge‐transfer photocycloaddition.

In 2020, an elegant supramolecular photocatalytic system via charge‐transfer excitation of a host‐guest complex was described by Iwasawa's group for the [4+2] cycloaddition reaction of anthracenes **91**.^[68]^ In particular, this supramolecular system which includes as host the macrocyclic boronic ester [2+2]*BTH‐F* decorated with electron‐deficient difluorobenzothiazole moieties, can generate the triplet state of guest molecule and thus, promoting the photocycloaddition of anthracenes with several dienes and alkenes in high yields. In detail, DFT calculations suggest that under visible light irradiation, the anthracene@[2+2]*BTH‐F* complex undergoes charge‐transfer excitation to induce a charge‐separated singlet state (Scheme 52). After rapid intersystem crossing, the triplet state of anthracene@[2+2]*BTH‐F* is reached, afterwards the triplet excited state of the anthracene is released into the solution readily available to execute the cycloaddition with diene **92**. Control experiments revealed, in fact, that the inclusion of the anthracene **91** was crucial to promote the cycloaddition reaction. This study may work as a seminal example harnessing supramolecular assembly to conduct selectively reactions under visible‐light irradiation.

## 5 Conclusions

In this Minireview, it has been described a concise set of strategies that successfully perform [2+2] and [4+2] photocycloadditions, lacking the use of metallic photocatalysts. Due to the attractiveness that these reactions represent from synthetic and mechanistic viewpoints, a vast literature is currently available. However, one of the main targets of this manuscript has been to highlight some of the “underdogs” on the subject using visible‐light photochemistry. The examples provided in this Minireview, not only demonstrate the robustness and importance that photocycloadditions mean in organic synthesis, but also should encourage the reader to go further in this same trend to tackle the remaining limitations. Additionally, it is clear that this research venture is far from ending, since innovative techniques as continuous flow processing, the involvement of heterogenous catalytic systems and host/guest supramolecular interactions, are joining as complementary tools, reinforcing the brilliantness of the topic.

## Conflict of interest

The authors declare no conflict of interest.

## Biographical Information

*Marina Sicignano received her M.S. in Chemistry from University of Salerno (Italy) in 2016. She obtained her PhD degree in 2020, which was focused on the development of new stereoselective routes using phase transfer catalysis conditions. She is currently a Postdoctoral Researcher in Photochemistry at Universidad Autónoma de Madrid, where her research aims to the discovery of new photochemical transformations in the synthesis of attractive chiral compounds*.

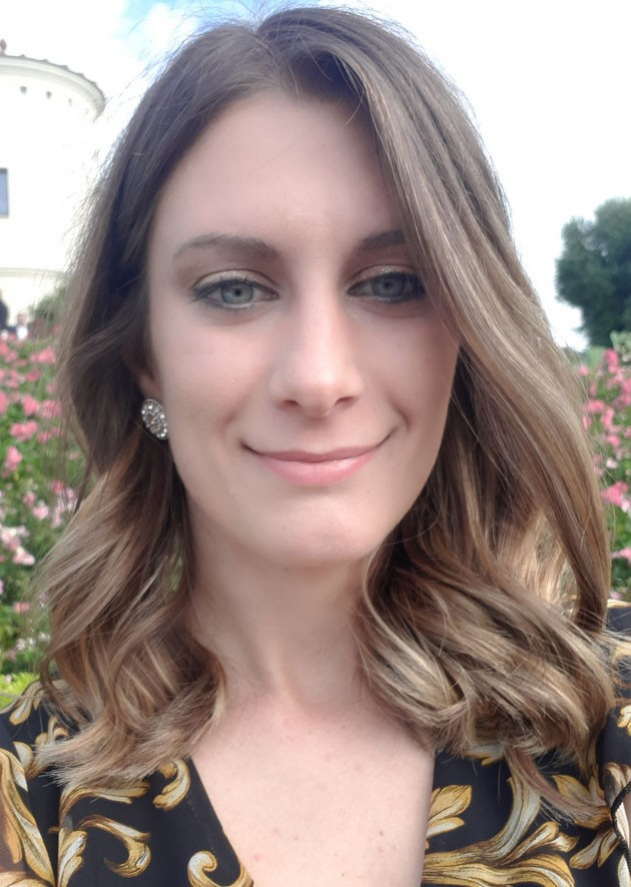

## Biographical Information

*Ricardo I. Rodriguez received his M.S. in Chemical Sciences (Organic Chemistry) from Universidad Nacional Autónoma de Mexico (2018), working on the development of asymmetric routes controlled by chiral sulfoxides. He is currently a third year PhD student at Universidad Autonoma de Madrid, conducting his research on the design of novel asymmetric photocatalyzed transformations, under the supervision of Prof. José Alemán*.

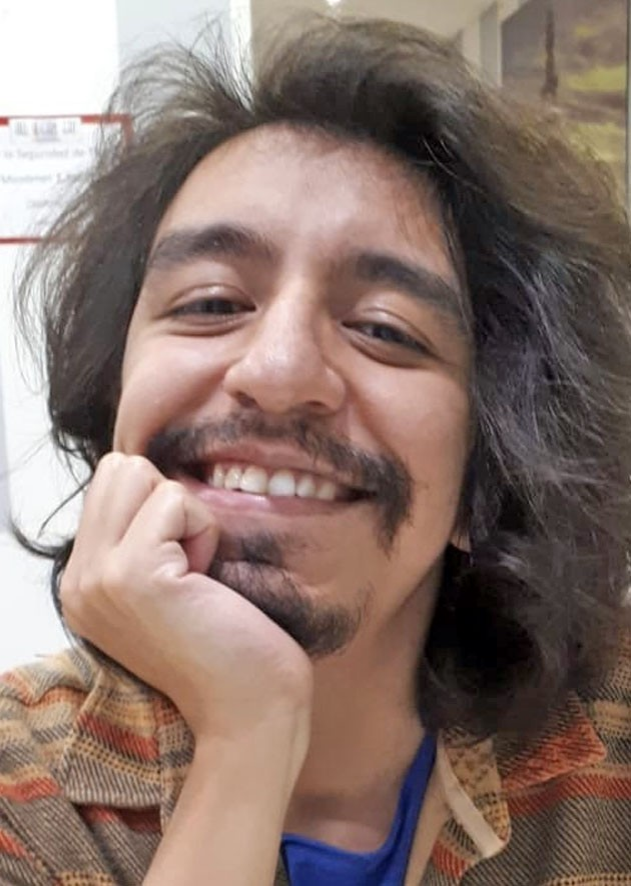

## Biographical Information

*José Alemán obtained his Ph.D. working on sulfur chemistry, under the supervision of Prof. García Ruano in 2005. In 2003, he spent six months in the laboratory of Prof. Albert Padwa at Emory University, Atlanta. Then, he carried out his postdoctoral studies (2006–2008) at the Center for Catalysis in Aarhus (Denmark) with Prof. Karl A. Jørgensen. He returned to Spain in 2009 as a Ramón y Cajal Researcher and is currently Professor at the Universidad Autónoma de Madrid (Spain). He is authour of 165 publications and his research interests include asymmetric synthesis and catalysis*.